# Receptor tyrosine kinase-like orphan receptor 1 inhibitor strictinin exhibits anti-cancer properties against highly aggressive androgen-independent prostate cancer

**DOI:** 10.37349/etat.2023.00192

**Published:** 2023-12-20

**Authors:** Vignesh Sivaganesh, Bela Peethambaran

**Affiliations:** The First Clinical Medical College of Lanzhou University, China; ^1^Department of Biology, Saint Joseph’s University, Philadelphia, PA 19104, USA; ^2^Department of Biomedical Sciences, Philadelphia College of Osteopathic Medicine, Philadelphia, PA 19131, USA

**Keywords:** Small molecule inhibitors, receptor tyrosine kinase-like orphan receptor 1, receptor tyrosine kinase, strictinin, prostate cancer, natural therapy, castration-resistant prostate cancer, oncology

## Abstract

**Aim:**

It is important to identify anti-cancer compounds that can inhibit specific molecular targets to eradicate androgen-receptor negative (AR^neg^), androgen-independent (AI) prostate cancer, which is an aggressive form of prostate cancer with limited treatment options. The goal of this study was to selectively target prostate cancer cells that have high levels of oncogenic protein Receptor tyrosine kinase-like orphan receptor 1 (ROR1) by using strictinin, a small molecule ROR1 inhibitor.

**Methods:**

The methods performed in this study include western blots, methyl thiazolyl tetrazolium (MTT) proliferation assays, phosphatidylserine apoptosis assays, apoptosis flow cytometry (Annexin V, caspase 3/7), migration scratch assays, Boyden chamber invasion assays, and cell cycle flow cytometry.

**Results:**

Strictinin was most lethal against PC3 [half-maximal drug inhibitory concentration (IC_50_) of 277.2 µmol/L], an AR^neg^-AI cell type that expresses the highest levels of ROR1. Strictinin inhibited ROR1 expression, downstream phosphatidylinositol 3-kinase (PI3K)-protein kinase B (AKT)-glycogen synthase kinase 3beta (GSK3β) pro-survival signaling, and epithelial-to-mesenchymal transition markers in PC3 cells. Additionally, strictinin decreased PC3 cell migration and invasion, while increasing S-phase cell cycle arrest. In AR^neg^-AI DU145 cells, strictinin inhibited ROR1 expression and modulated downstream AKT-GSK3β signaling. Furthermore, strictinin exhibited anti-migratory, anti-invasive, but minimal pro-apoptotic effects in DU145 cells likely due to DU145 having less ROR1 expression in comparison to PC3 cells. Throughout the study, strictinin minimally impacted the phenotype of normal prostatic epithelial cells RWPE-1 (IC_50_ of 658.5 µmol/L). Strictinin was further identified as synergistic with docetaxel [combination index (CI) = 0.311] and the combination therapy was found to reduce the IC_50_ of strictinin to 38.71 µmol/L in PC3 cells.

**Conclusions:**

ROR1 is an emerging molecular target that can be utilized for treating prostate cancer. The data from this study establishes strictinin as a potential therapeutic agent that targets AR^neg^-AI prostate cancer with elevated ROR1 expression to reduce the migration, invasion, cell cycle progression, and survival of prostate cancer.

## Introduction

Prostate cancer is a disease that has a high prevalence and mortality among men. The severity of the cancer is stratified by risk, which is based on the Gleason grade, prostate-specific antigen (PSA) level, and clinical staging [1]. Unlike breast cancer which can be classified into subtypes based on molecular heterogeneity, prostate cancer has no such classification and is generally treated with observation, prostatectomy, or radiation if local, and chemotherapy and hormone therapy if the cancer is advanced or metastatic [1]. Recently, prostatic acid phosphatase, polyadenosine-diphosphate-ribose polymerase (PARP), prostate-specific membrane antigen, and programmed cell death protein 1 (PD1) targeting therapies have been approved for certain forms of metastatic prostate cancer [2–4]. These limited forms of targeted therapy may have different efficacies in patients due to tumor heterogeneity [5–11]. Moreover, while these forms of therapy are used for the treatment of metastatic castration-resistant prostate cancer (CRPC), none of these therapies specifically target prostate cancers that have androgen receptor-negative profiles.

Studies on androgen synthesis inhibitors (e.g., abiraterone) and androgen receptor antagonists (e.g., enzalutamide) have found that these forms of hormone therapy are increasing the incidence of androgen receptor negative (AR^neg^), androgen-independent (AI) prostate cancers [12–14]. These cancers are a form of CRPC and are especially hard to treat since the androgen receptor is absent and oncogenic signaling is activated through many other modalities [12]. Better molecular markers are required to specifically identify and eradicate prostate cancers that exhibit resistance to hormone therapy due to a lack of androgen-receptor (AR).

One possible marker to be considered is the receptor tyrosine kinase-like orphan receptor 1 (ROR1), which is an oncofetal receptor tyrosine kinase (RTK)-like protein that is highly expressed in embryogenesis but is largely absent in mature tissue [15–17]. ROR1 is also uniquely overexpressed in a wide variety of cancers, including prostate cancer [15]. ROR1, like other RTKs, dimerizes upon ligand (i.e., Wnt5a) binding to elicit the activation of multiple downstream oncogenic pathways [18, 19]. The canonical Wnt signaling pathway activates Frizzled (FZD) and low-density lipoprotein receptor-related protein (LRP) 5/6 co-receptors, which represses the β-catenin destruction complex and allows for β-catenin to regulate gene expression [20–22]. ROR1 regulates β-catenin independent pathways, which is defined as non-canonical Wnt signaling, and activates downstream oncogenic cascades like the phosphatidylinositol 3-kinase (PI3K)-protein kinase B (AKT) and rat sarcoma-mitogen-activated protein kinase (RAS-MAPK) pathways [20, 22]. ROR1 has many pro-survival functions in cancer such as enhancing cell proliferation, epithelial-to-mesenchymal transition (EMT), metastasis, cancer stemness, and therapy resistance [22]. Studies have shown that inhibiting ROR1 effectively targets many forms of ROR1-expressing cancers [23–25]. Hence, prostate cancers that exhibit elevated levels of ROR1 may be specifically targeted by established forms of therapy such as immunotherapy or small molecule inhibitors.

Small molecule inhibitors are known to be effective in cancer therapeutics. One potential untapped resource of small molecule inhibitors can be obtained through extraction from medicinal plants and teas. One such natural compound, known as strictinin, has medicinal properties for a variety of ailments [26–29]. Our lab has previously shown that strictinin can selectively inhibit ROR1 in triple-negative breast cancer (TNBC) [29]. Corroborating these results was a reduction in oncogenic signaling pathways as well as migration, invasion, and anti-apoptosis in aggressive TNBC cells [29]. Furthermore, our previous research identified that ROR1 was an important molecular marker for AR^neg^-AI prostate cancer cell lines (PC3 and DU145) [30]. Hence, we wanted to explore whether ROR1 inhibitor strictinin can selectively target aggressive AR^neg^-AI prostate cancer. Strictinin has not been previously explored as a treatment to kill aggressive prostate cancers.

In this study, we identify that strictinin inhibits ROR1 and modulates the subsequent downstream oncogenic signaling cascade in AR^neg^-AI prostate cancer cells. Strictinin elicited selective cytotoxicity and apoptosis of PC3 cells. Based on our previous research that identified ROR1 expression to be most upregulated in the PC3 (AR^neg^-AI cancer) cell line, we further investigated whether strictinin suppressed ROR1 and downstream cancerous signaling in PC3 cells [30]. We found that strictinin reduced ROR1 expression and downstream PI3K-AKT-glycogen synthase kinase 3beta (GSK3β) signaling in PC3 cells. By inhibiting this central oncogenic signaling cascade in PC3, strictinin also suppressed further downstream pathways related to anti-apoptosis, EMT, migration, and invasion. These molecular results were supported by reduced migration, stunted invasion, and an increase in S-phase cell cycle arrest of PC3 cells. Strictinin had minimal impact on RWPE-1 cells (normal prostatic epithelium) and moderate phenotypic anti-cancerous effects on DU145 cells (AR^neg^-AI cancer), which is most likely due to the minimal and moderate expression of ROR1 found in RWPE-1 cells and DU145 cells, respectively [30]. We also identified that strictinin inhibited ROR1 expression and modulated the downstream AKT-GSK3β signaling axis in DU145 cells. Our results indicate that strictinin can be selectively lethal, anti-migratory, and anti-invasive to AR^neg^-AI prostate cancer cells by modulating ROR1-mediated AKT-GSK3β signaling. Additionally, strictinin can be synergistic with docetaxel. The natural compound strictinin, as well as the emerging molecular target ROR1, have marked potential for the clinical treatment of aggressive AR^neg^-AI prostate cancers.

## Materials and methods

### Cell culture

PC3 [CRL-1435, American Type Culture Collection (ATCC)], RWPE-1 (CRL-11609, ATCC), and DU145 (HTB-81, ATCC) cells were maintained in culture medium and subcultured as per ATCC recommendations. These cell lines were bought from ATCC and tested for mycoplasma infection. PC3 cells were grown in Ham’s F-12K (Kaighn’s) medium (#21127022, Thermofisher, USA) that was supplemented with 10% of fetal bovine serum (FBS, #FBS-500, CPS Serum, USA). RWPE-1 cells were grown in keratinocyte serum-free medium (KSFM, #17005042, Thermofisher, USA) that was supplemented with 10% of FBS. The 500 mL bottles of KSFM were also supplemented with 25 mg bovine pituitary extract and 2.5 µg of human recombinant epidermal growth factor that was packaged together with the media. DU145 cells were grown in Eagle’s minimum essential medium (EMEM, #30-2003, ATCC) that was supplemented with 10% of FBS.

### Preparation of strictinin and vehicle control for cell treatment

Five milligrams of the strictinin compound (#NH026102, Nacalai USA) were dissolved in dimethyl sulfoxide (DMSO, #J66650.AP, Thermofisher, USA) to achieve a concentration of 15.76 mmol/L or 197.02 mmol/L. Then, strictinin was mixed with the appropriate media required for PC3, RWPE-1, and DU145 to obtain the desired concentrations (up to 1,000 µmol/L). Cells were also given 0.1% to 0.5% DMSO (non-toxic to cells) mixed with the appropriate media, which served as a vehicle control.

### Preparation of docetaxel and vehicle control for cell treatment

Docetaxel (#01885, Sigma, USA) was dissolved in DMSO to create a stock solution and then mixed with media to generate a specified concentration (up to 2,500 nmol/L). A control solution of DMSO in media was generated for each experiment to match the percentage of DMSO in conditions treated with the highest concentration of docetaxel. DMSO concentrations did not exceed 0.5% of the cell culture media for treatment and control conditions.

### Antibody list for western blot

ROR1 (#16540S, Cell signaling, USA); AKT (#9272S, Cell signaling, USA); phosphorylated-AKT-ser473 [p-AKT-ser473 (#4060S, Cell signaling, USA)]; GSK3β (#12456S, Cell signaling, USA); p-GSK3β-ser9 (#5558S, Cell signaling, USA); Twist1 (#69366S, Cell signaling, USA); matrix metalloproteinase 9 (MMP9, #13667S, Cell signaling, USA); Snail (#3895S, Cell signaling, USA); glyceraldehyde-3-phosphate dehydrogenase (GAPDH, #C2819, scbt, USA); HRP conjugated Goat anti-rabbit secondary (#7074S, Cell signaling, USA); HRP conjugated Goat anti-mouse secondary (#1706516, Biorad, USA).

### Western blot

PC3 and DU145 cells were treated with strictinin or vehicle control. After 24 h to 48 h of treatment, cell culture flasks were washed with ice-cold phosphate buffer solution (PBS, 0.01 mol/L, #10-010-023, Thermofisher, USA) and then scraped while submerged in PBS to obtain the cells. Cells were centrifuged to aspirate the PBS and isolate the cell pellets. Cells were lysed using radioimmunoprecipitation assay (RIPA) buffer (#89900, Thermofisher, USA) containing protease inhibitor (0.01 mol/L, #78430, Thermofisher, USA) and ethylenediaminetetraacetic acid (EDTA, #78430, Thermofisher, USA), followed by centrifugation (14,000 *g* for 15 min) to isolate the protein. Protein concentrations of PC3 and DU145 protein lysates, which were harvested from varying treatment conditions, were obtained using either the Pierce 660 nm Protein Assay (#22660, ThermoFisher, USA) or the Pierce BCA protein assay kit (#23225, ThermoFisher, USA). Proteins were diluted at a 1:1 ratio with laemmli buffer (0.02 mol/L, #1610737EDU, Biorad, USA) and denatured by heating the samples at 100℃ for 5–10 mins. Then, equal amounts of protein (15–20 µg) were loaded into a Tris-Glycine gel (4–15%) and separated at 100 V for 85 min. After separation, proteins were transferred to a nitrocellulose membrane using the iBlot dry transfer system (iBlot 1 or 2, Thermofisher, USA). Then, proteins of interest were probed using a 1:1,000 dilution of primary antibody [5% milk or 5% bovine serum albumin (BSA)], followed by a 1:3,000 dilution of anti-rabbit or anti-mouse secondary antibodies that corresponded to the species within which the primary antibody was produced. Proteins of interest were visualized by adding a luminol detection reagent, followed by imaging with a Biorad chemiluminescence imager (12003154, ChemiDoc imaging system). All band intensities were quantified and normalized to GAPDH using ImageJ.

### Methyl thiazolyl tetrazolium cell viability assay

On day 1, PC3 and RWPE-1 cells were seeded in 96-well culture plates (#3904, Corning, USA) at a certain cell density per well (e.g., 10,000 cells/well) to maintain uniformity throughout the cell viability assay. After cell attachment occurred on day 2, cells were treated with strictinin in fresh media at 1,000 µmol/L, 500 µmol/L, 250 µmol/L, 125 µmol/L, 62.5 µmol/L, and 31.25 µmol/L. These concentrations were achieved through serial dilution. After 24–72 h post-treatment, the strictinin-containing media was aspirated and fresh media was introduced along with methyl thiazolyl tetrazolium (MTT) dye. Cells were incubated in MTT dye for 2–4 h at 37℃ followed by adding DMSO and reading the plates (Spectramax ABS, Molecular Devices, USA) at an absorbance value of 540 nm. Cell viability was calculated as the ratio of absorbance readings from treated wells to non-treatment or vehicle control wells.

### Phosphatidylserine apoptosis assay

RWPE-1 cells were plated at equal densities (e.g., 25,000–50,000 cells) in a black-walled, clear bottom, 96-well plate (#3904, Corning, USA) and treated with 250 µmol/L strictinin or vehicle control. After 72 h of treatment, cells were incubated with fresh media and orange fluorescent apopxin working solution at a 1:1 ratio as per the protocol’s instructions (#22794, AAT bioquest, USA). After incubation at room temperature for 1 h, fluorescence intensities were read with a Tecan microplate reader (Infinite^®^200 PRO, Tecan infinite 200 multimode microplate reader) at an excitation and emission of 540 nm and 590 nm. The fluorescence intensity values indicated how much phosphatidylserine was present on the cell surface.

### Annexin V and caspase 3/7 apoptosis assay

PC3 and DU145 cells were treated with strictinin or vehicle control (DMSO). Then, the cells were prepared for flow cytometry using the Muse caspase 3/7 kit (#MCH1000108, Luminex, USA). Separately, PC3 cells were also prepared for flow cytometry after treatment using the Muse Annexin V kit (#MCH100105, Luminex, USA). Then, samples were run through the Muse flow cytometer (Muse cell analyzer, Luminex, USA), which provided the percentage of cells in different stages of apoptosis.

### Wound healing (scratch) assay

Cells were seeded into 6-well dishes and were grown to near 100% confluence prior to generating the wounds. PC3, DU145, and RWPE-1 cells were subjected to 250 µmol/L strictinin, 125 µmol/L strictinin, or vehicle control conditions. At 0 h, scratches were generated within the wells using a pipette tip to mimic the formation of a wound, and fresh media containing the desired concentration of strictinin or DMSO was introduced into the wells. Using an inverted phase contrast microscope (SKU 11526208, Leica DMi1 inverted phase contrast), images of the cells were obtained at 0 h and 24 h after the scratches were generated. ImageJ was used to quantify the sizes of the wounds. The percent wound healed was calculated as a ratio of the scratch area at 24 h to the area of the scratch at 0 h.

### Invasion assay

A Boyden chamber (#3455-024-K, Trevigen, USA) consists of a basement membrane-containing insert that can float on media within a 24-well plate (#3524, Corning, USA). The insert itself is considered the top chamber where cells can be seeded, while the well is considered the bottom chamber and contains media fully supplemented with serum that acts as a chemoattractant. PC3, DU145, and RWPE-1 cells were serum starved for 24 h prior to being seeded in the top chamber. Shortly after, cells were treated with 250 µmol/L strictinin or vehicle control in fresh serum-starved media. FBS—containing media was added to the bottom chamber. Over the course of 24 h, cells invaded through the basement membrane and migrated to the bottom chamber containing the fully supplemented media. At the 24 h mark, the cells that invaded through and attached to the other side of the basement membrane were stained using crystal violet, imaged, and quantified using ImageJ. Multiple non-overlapping regions of the Boyden chamber inserts were imaged for each treatment condition in order to capture a wide array of invaded cells for data analysis.

### Cell cycle analysis

PC3 and RWPE-1 cells were treated with 250 µmol/L strictinin, 125 µmol/L strictinin, or vehicle control for 24 h. Then, cells were processed using the Muse Cell Cycle kit (#MCH100106, Luminex, America) and ran through the Muse flow cytometer to obtain the percentage of cells in various stages of the cell cycle.

### Combination drug treatment MTT proliferation assay

PC3 cells were plated at a uniform density per well in a 96-well plate. The cells were treated with a combination of strictinin and docetaxel (72 h) which were both serially diluted to achieve a range of combination concentrations. Then, after processing the plate via the addition of MTT dye and DMSO, the plate was read at a wavelength of 540 nm to obtain absorbance values for each well. Cell viability was calculated as the ratio of absorbance in treated wells to control wells. The synergistic effect of strictinin and docetaxel was evaluated through isobologram analysis, identifying a statistically significant difference in half-maximal drug inhibitory concentration (IC_50_) between monotherapy and combination therapy, and combination index (CI) analysis [31–33].

### Statistical analysis

Microsoft Excel was used to process raw data prior to being utilized in Graphpad Prism (La Jolla California USA, www.graphpad.com). The experiments were analyzed via *t*-test or one-way ANOVA with Tukey’s multiple comparisons post-hoc test. The MTT cell viability experiments were analyzed via linear regression and comparison of fits performed on GraphPad Prism. The Chou-Talalay isobologram and CI method were utilized to evaluate synergy [33]. GraphPad Prism was used to perform statistical analysis and obtain *P*-values for the experiments [^****^ *P* value < 0.0001; ^***^ *P* value < 0.001; ^**^ *P* value < 0.01; ^*^ *P* value < 0.05; not significant (ns)].

## Results

### Strictinin promotes apoptosis of PC3 cells

**Figure 1:**
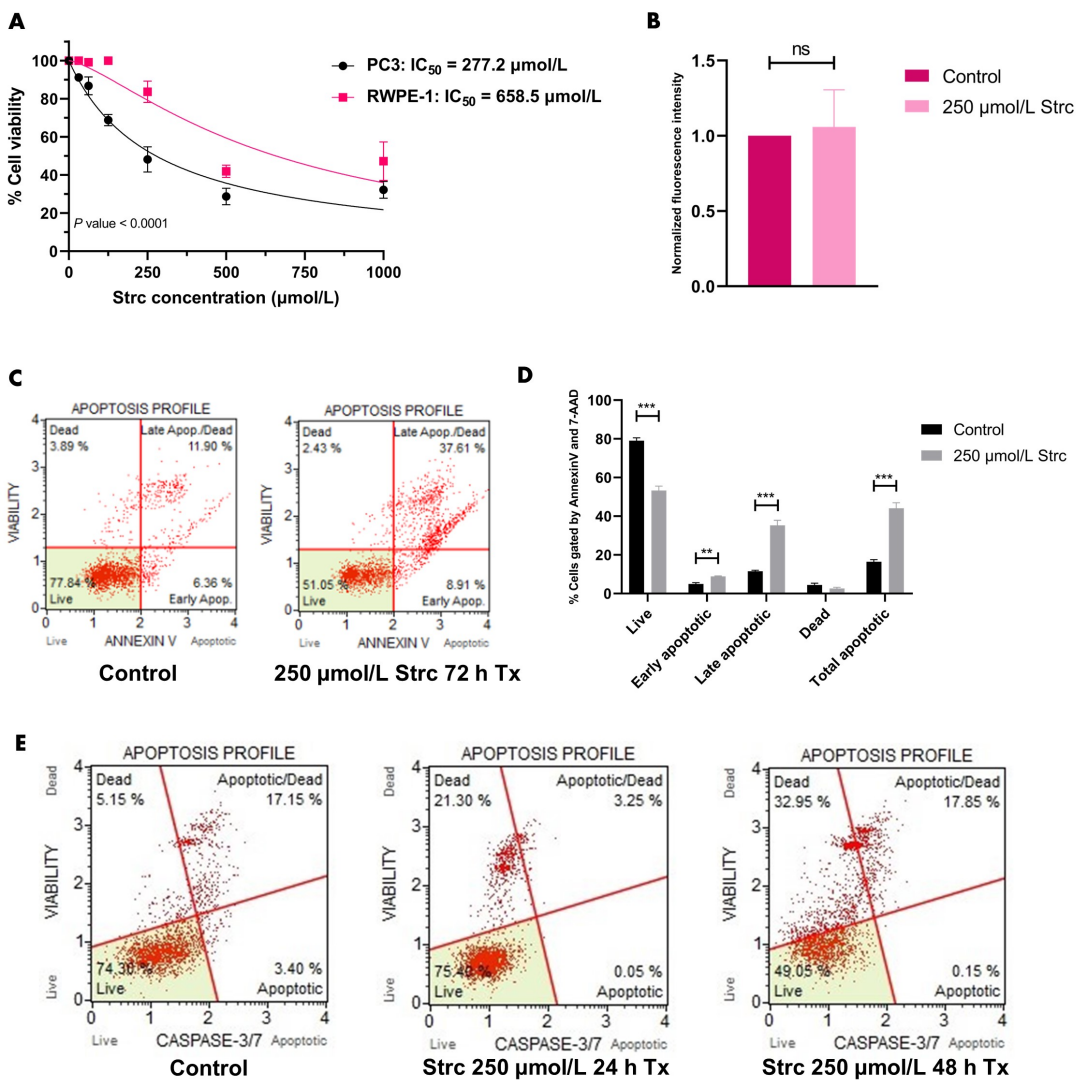
Strictinin is selectively cytotoxic and lethal to PC3 cells. (A) Assessment of strictinin concentrations on cell viability of PC3 and RWPE-1 after 72 h of treatment; (B) Annexin V phosphatidylserine expression of RWPE-1 cells treated with control or strictinin for 72 h. Fluorescence intensity data were normalized to controls; (C) representative data of Muse flow cytometry Annexin V apoptosis profile. Cells were gated based on the intensity of Annexin V and 7-AAD fluorescence; (D) quantification of Annexin V apoptosis profile on treated PC3 cells (control or 250 μmol/L strictinin for 72 h). Cells were sorted into live, early apoptosis, late apoptosis, dead, or total apoptosis based on the intensity of Annexin V and 7-AAD fluorescence; (E) Muse flow cytometry apoptosis profile on treated PC3 cells (control, 250 μmol/L strictinin for 24 h, or 250 μmol/L strictinin for 48 h). Cells were sorted based on caspase 3 and caspase 7 fluorescence intensity. *N* ≥ 3. ^***^ *P* value < 0.001; ^**^ *P* value < 0.01; strc: strictinin; Tx: treatment; Apop: apoptotic

To determine if strictinin elicited cytotoxicity and apoptosis in aggressive PC3 prostate cancer cells while minimally affecting RWPE-1, a 72 h proliferation assay on PC3 and RWPE-1 cells at different concentrations of strictinin was performed. The results showed that the IC_50_ of strictinin in PC3 (277.2 μmol/L) was about 2.4-fold lower than in RWPE-1 (Figure 1A). Next, Annexin V fluorescence intensity was assessed to determine the extent of apoptosis in RWPE-1 cells treated with 250 μmol/L of strictinin (near IC_50_ value in PC3 cells) for 72 h. The analysis revealed that strictinin did not significantly upregulate the early apoptotic marker phosphatidylserine, meaning that strictinin did not induce significant apoptotic signaling in RWPE-1 cells after 72 h of 250 μmol/L strictinin treatment (Figure 1B). This led us to investigate strictinin-induced apoptosis in PC3 cells. Strictinin caused significant apoptosis of PC3 cells after 72 h of 250 μmol/L strictinin treatment, which was evaluated based on Annexin V (phosphatidylserine) and late apoptotic marker 7-aminoactinomycin D (7-AAD) protein expression (Figure 1C and D). Furthermore, strictinin induced pro-apoptotic caspase 3 and caspase 7 expression, which increased the percentage of apoptotic and dead PC3 cells in the 48 h strictinin-treated condition in comparison to the control (Figure 1E and Figure S1). Taken together, the results indicate that strictinin is selectively cytotoxic to PC3 cells after 72 h of treatment and induces significant apoptosis after 48 h of treatment (250 μmol/L). Additionally, strictinin, at a near PC3 IC_50_ value (250 μmol/L), is non-lethal to RWPE-1 cells (Figure 1A and B).

### Strictinin inhibits ROR1-mediated oncogenic signaling in PC3 cells

**Figure 2:**
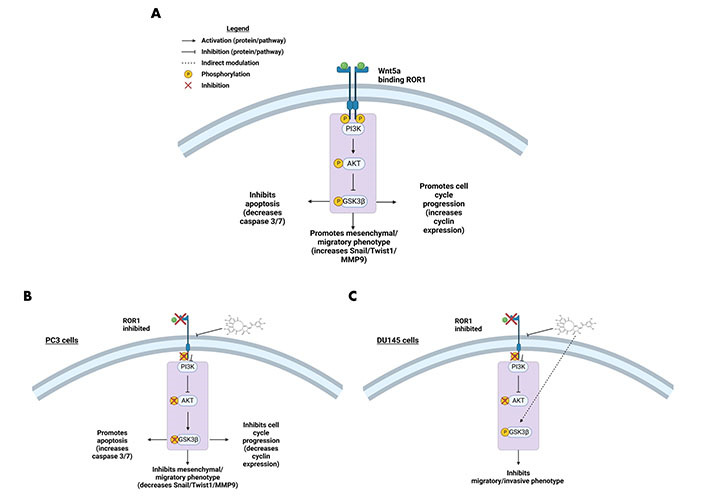
ROR1 and the downstream oncogenic signaling pathway are affected by strictinin. (A) Illustration of active ROR1 pathway. ROR1 leads to increased p-AKT and p-GSK3β, which promotes downstream oncogenic signaling pathways; (B) modulation of the ROR1 pathway by strictinin in PC3 cells. ROR1 inhibition diminishes p-AKT and p-GSK3β, which increases apoptosis and represses cell cycle progression, invasion, and migration in PC3 cells; (C) modulation of the ROR1 pathway by strictinin in DU145 cells. ROR1 inhibition diminishes p-AKT and strictinin increases the phosphorylation of GSK3β, which is likely a contributing molecular mechanism by which strictinin suppresses the migration and invasion of DU145 cells. The figure was created with Biorender.com

**Figure 3:**
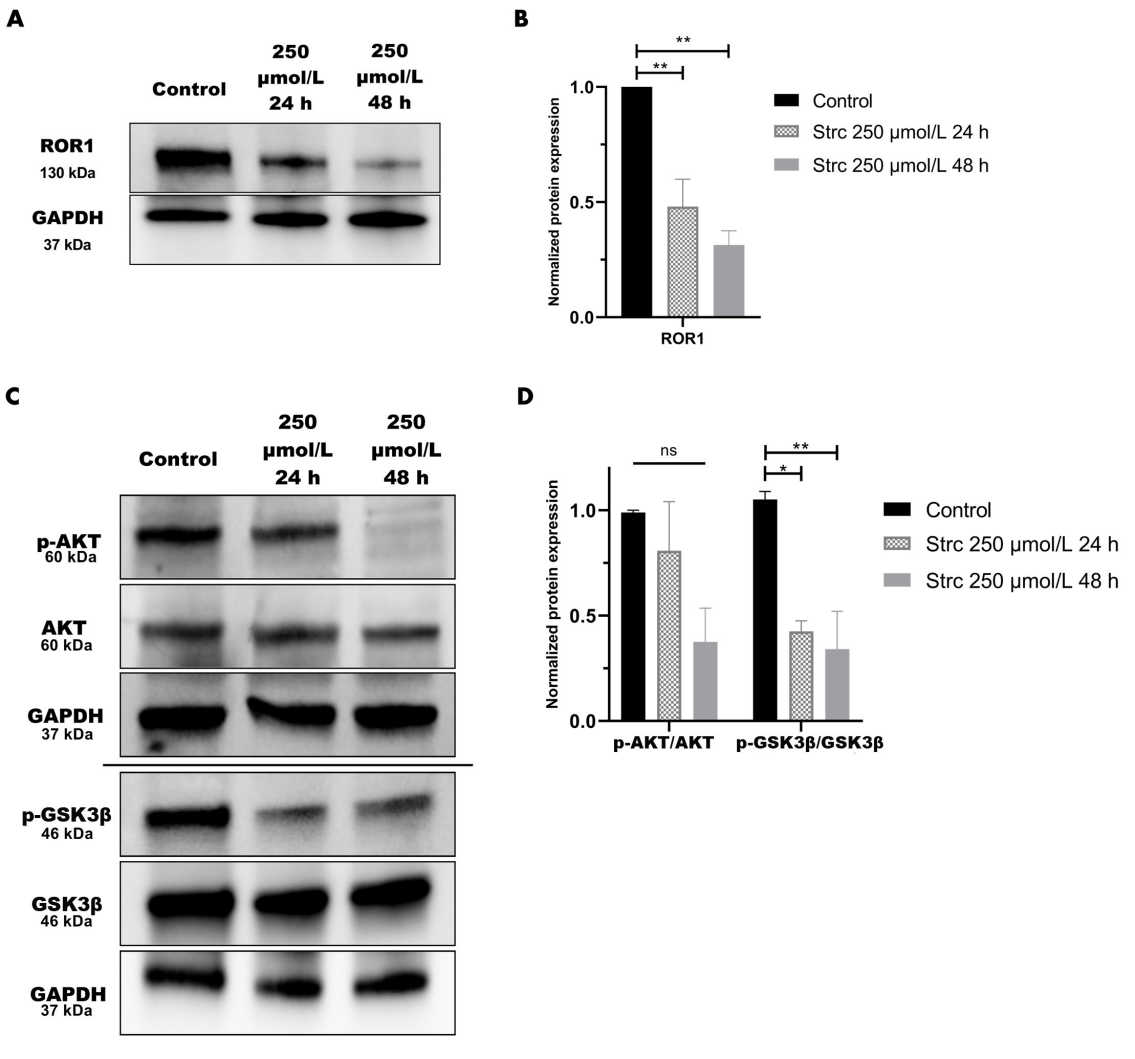
Strictinin inhibits ROR1-mediated oncogenic signaling in PC3 cells. (A) Immunoblot on PC3 cells treated with control, 250 µmol/L strictinin for 24 h, or 250 µmol/L strictinin for 48 h. ROR1 is visualized; (B) quantification of ROR1 protein band normalized to GAPDH. Data are represented as the mean ± standard error of the mean (SEM) from three independent western blot experiments; (C) immunoblot on PC3 cells treated with control, 250 µmol/L strictinin for 24 h, or 250 µmol/L strictinin for 48 h. AKT, p-AKT-ser473, GSK3β, p-GSK3β-ser9 visualized; (D) quantification of p-AKT/AKT and p-GSK3β/GSK3β protein bands normalized to GAPDH. Data are represented as the mean ± SEM from three independent western blot experiments. *N* = 3. ^**^ *P* value < 0.01; ^*^ *P* value < 0.05

RTK-like protein ROR1, upon ligand binding and activation, is known to elicit downstream PI3K-AKT-GSK3β pro-survival signaling (Figure 2) [22]. AKT and GSK3β are often phosphorylated in dysregulated cancer signaling that arises from aberrant RTK activity [34]. Prior research in our lab identified a strong interaction between ROR1 and strictinin through *in silico* docking analysis and isothermal calorimetry experiments [29]. Since our prior studies suggested that strictinin interacts with ROR1 and that ROR1 expression was most upregulated in PC3 cells, we sought to explore whether strictinin suppresses ROR1 and downstream cancerous signaling in PC3 cells. We identified that the expression of ROR1, p-AKT-ser473, and p-GSK3β-ser9 was diminished, especially in the 48 h strictinin treatment condition in comparison to the control (Figure 3). Together, these data suggest that strictinin suppresses the expression of ROR1, which leads to the inhibition of downstream PI3K-AKT-GSK3β signaling in PC3 cells (Figure 2B). Strictinin likely exerts a selective lethal effect on PC3 cells by inhibiting ROR1 and downstream p-AKT to induce pro-apoptotic signaling (Figure 2B, Figure 3A and C).

### Strictinin inhibits the migration and invasion of PC3 cells

**Figure 4:**
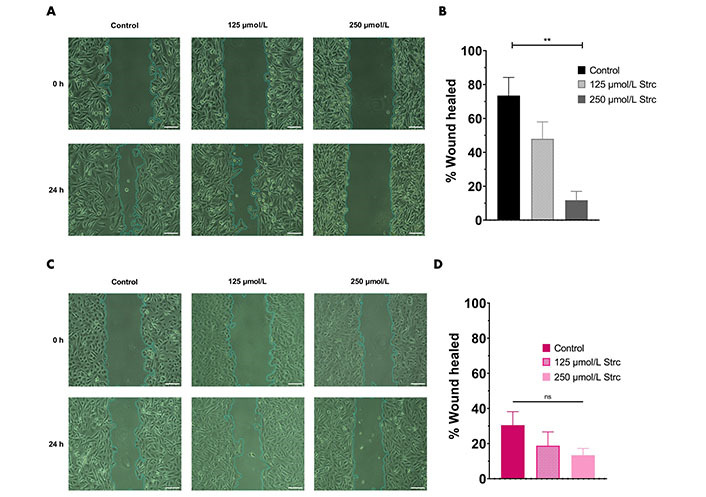
Strictinin inhibits the migration of PC3 cells. (A) Wound healing (migration) assay on PC3 cells treated with control, 125 µmol/L strictinin, or 250 µmol/L strictinin for 24 h. All images were taken at 10× magnification. Scale bar represents 200 pixels; (B) PC3 wound healing assays quantified as percentage wound healed to evaluate the extent of migration in treated PC3 cells; (C) wound healing assay on RWPE-1 cells treated with control, 125 µmol/L strictinin, or 250 µmol/L strictinin for 24 h. All images were taken at 10× magnification. Scale bar represents 200 pixels; (D) RWPE-1 wound healing assays quantified. *N* ≥ 3. ^**^ *P* value < 0.01

**Figure 5:**
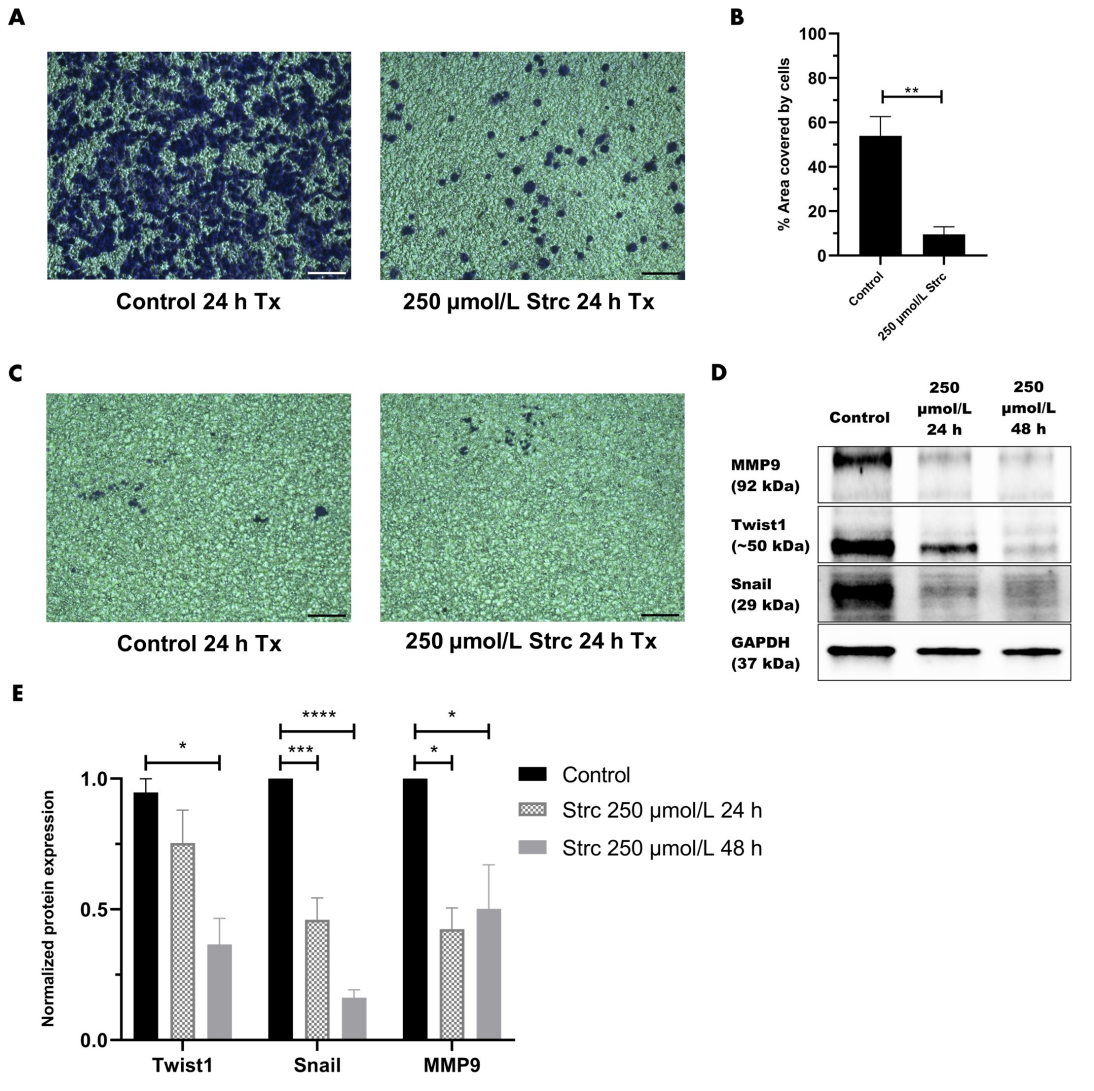
Strictinin inhibits the invasion of PC3 and the expression of pro-migratory and pro-invasive proteins in PC3 cells. (A) Bright-field image of invasive, crystal-violet stained PC3 cells after 24 h control or strictinin treatment. All images were taken at 10× magnification. Scale bar represents 200 pixels; (B) quantification of PC3 cell invasion represented as the percentage area covered by crystal-violet stained cells to quantify the extent of invasion in treated PC3 cells; (C) bright field image of invasive, crystal-violet stained RWPE-1 cells after 24 h control or strictinin treatment. All images were taken at 10× magnification. Scale bar represents 200 pixels; (D) immunoblot of MMP9, Twist1, and Snail on PC3 cells treated with control, 250 µmol/L strictinin for 24 h, or 250 µmol/L strictinin for 48 h; (E) quantification of MMP9, Twist1, and Snail protein bands normalized to GAPDH. Data are represented as the mean ± SEM from three independent western blot experiments. *N* = 3. ^****^ *P* value < 0.0001; ^***^ *P* value < 0.001; ^**^ *P* value < 0.01; ^*^ *P* value < 0.05. ~: approximate

ROR1 can promote the migration and invasion of cancer [29, 35, 36]. Therefore, we predicted that strictinin should suppress the migration and invasion of prostate cancer cells (Figures 4 and 5). To investigate whether strictinin impacted the migration and invasion of PC3, the cells underwent a 24 h scratch assay with control, 125 μmol/L of strictinin, or 250 μmol/L of strictinin treatment (Figure 4A). PC3 cells were also subjected to 24 h Boyden chamber invasion studies (Figure 5A). PC3 cells migrated and invaded substantially less in the 250 μmol/L strictinin-treated condition in comparison to the control (Figure 4A and B, Figure 5A and B). RWPE-1 cells also went through 24 h migration and invasion studies under the same treatment conditions as PC3 (Figure 4C and Figure 5C). The migration and invasion of RWPE-1 cells, which was minimal overall, did not significantly differ between control and strictinin-treated conditions (Figure 4C and D, Figure 5C). We also found that the 24 h cell viability of PC3 and RWPE-1 cells was not significantly changed at concentrations as high as 250 μmol/L of strictinin, which confirms that cell viability did not impact the migration and invasion studies (Figure S2A and B). The activation of the PI3K-AKT-GSK3β pathway downstream of ROR1 is known to promote EMT by increased expression of mesenchymal markers Snail, Twist1, and MMP9, which all play a significant role in the migration and invasion of cancer (Figure 2A) [37–40]. Since prior experiments revealed that strictinin suppressed ROR1 and downstream p-AKT/p-GSK3β signaling, we wanted to assess whether strictinin also mitigated the expression of mesenchymal markers Snail, Twist1, and MMP9 in PC3 cells. We determined that strictinin inhibited the expression of Twist1, Snail, and MMP9 in PC3 cells, especially at 250 μmol/L of strictinin for 48 h of treatment (Figure 5D and E). These results strongly suggest that strictinin hinders the migration and invasion of PC3 cells while minimally impacting RWPE-1 cell phenotype. Additionally, these findings reveal that the inhibition of ROR1-mediated AKT-GSK3β signaling by strictinin suppresses the expression of Twist1, Snail, and MMP9 in PC3 cells. The reduction in Twist1, Snail, and MMP9 expression likely suggests that the cells are undergoing a mesenchymal-to-epithelial transition (MET), which diminishes the mesenchymal migratory and invasive characteristics seen in aggressive PC3 prostate cancer cells (Figure 2B).

### PC3 cells are S-phase arrested upon strictinin treatment

**Figure 6:**
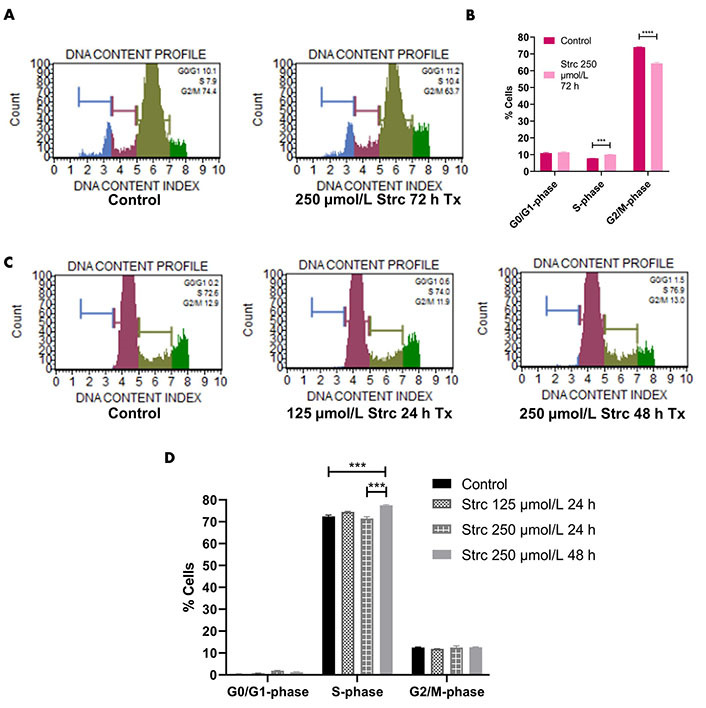
Strictinin arrests PC3 in the S-phase of the cell cycle. (A) Muse flow cytometry of RWPE-1 cells treated with control or 250 µmol/L strictinin for 72 h. Cells were categorized into different phases of the cell cycle based on propidium iodide-stained DNA; (B) quantification of cell cycle flow cytometry on treated RWPE-1 cells; (C) Muse flow cytometry of PC3 cells treated with control, 125 µmol/L strictinin for 24 h, or 250 µmol/L strictinin for 48 h. Cells were categorized into different phases of the cell cycle based on propidium iodide-stained DNA; (D) quantification of cell cycle flow cytometry on treated PC3 cells. *N* = 3. ^****^ *P* value < 0.0001; ^***^ *P* value < 0.001

Since the PI3K-AKT-GSK3β pathway influences cell cycle dynamics, we wanted to determine whether strictinin affected prostate cancer cell cycle regulation (Figure 2A) [41–55]. RWPE-1 cells displayed an S-phase arrest when treated with 250 μmol/L strictinin for 72 h (Figure 6A and B). Meanwhile, PC3 cells displayed an S-phase cell cycle arrest when treated with 250 μmol/L strictinin for 48 h (Figure 6C and D). Both prostate cancer PC3 and normal prostate epithelium RWPE-1 exhibited cell cycle arrest upon treatment with strictinin (Figure 6). However, strictinin-induced S-phase arrest was higher in magnitude in PC3 cells (~5%) *vs.* RWPE-1 cells (~2%). Additionally, RWPE-1 cells exhibited a ~2% S-phase arrest after being treated for a longer period of time than the PC3 cells (72 h *vs.* 48 h). Our findings demonstrate that strictinin causes an S-phase cell cycle arrest that is more selective to AR^neg^-AI PC3 cells than RWPE-1 cells (Figure 2B).

### Strictinin and docetaxel display synergism in PC3 cells

**Figure 7:**
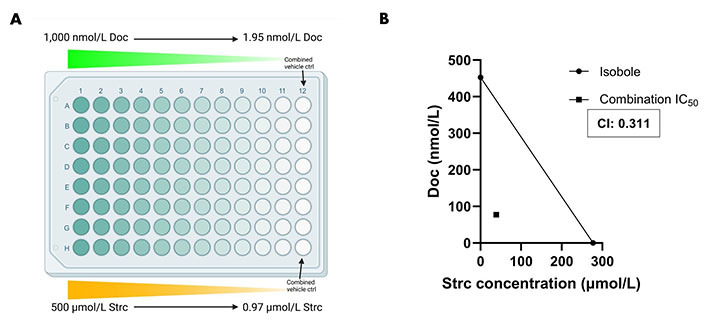
Combination strictinin and docetaxel treatment exhibits synergism and decreases the IC_50_ of both compounds in PC3 cells. (A) Illustration of combination treatment plate map (the figure was created with Biorender.com). Both docetaxel and strictinin were introduced at the highest concentration and serially diluted; (B) the isobole is drawn from the IC_50_ of docetaxel and strictinin when used individually. The square dot depicts the combination IC_50_. The CI for strictinin and docetaxel was determined to be 0.311. *N* ≥ 3. Doc: docetaxel

Docetaxel is a common chemotherapeutic agent used for the treatment of prostate cancer [46, 47]. However, it can display severe side effects that can affect a patient’s treatment outcome [46, 48, 49]. Therefore, we explored the possibility that a strictinin and docetaxel combination could be synergistic and lower the concentration required of both compounds to attain significant cytotoxicity in PC3 cells. A range of strictinin and docetaxel concentrations were simultaneously introduced to PC3 cells, followed by an assessment of cell viability after 72 h of combination treatment (Figure 7A). Our experiment revealed that the combination treatment significantly decreased the IC_50_ and caused a left shift in the combination therapy cell viability curve when compared to monotherapy (Figure S3A and B). The IC_50_ of strictinin was reduced by about 7-fold (277.2 μmol/L to 38.71 μmol/L) and docetaxel by about 6-fold (452.7 nmol/L to 77.43 nmol/L) when used in combination (Figure S3A and B). The combination IC_50_ was also below the isobole, with a CI of 0.311 (Figure 7B). The isobologram, a CI less than 1, and a significant reduction in IC_50_ of both compounds when used in combination all demonstrate that docetaxel and strictinin work synergistically in PC3 cells [31–33]. These data demonstrate that lower amounts of both strictinin and docetaxel can be used in combination to generate significant lethality of aggressive prostate cancer cells.

### Strictinin modulates ROR1-mediated oncogenic signaling in DU145 cells

**Figure 8:**
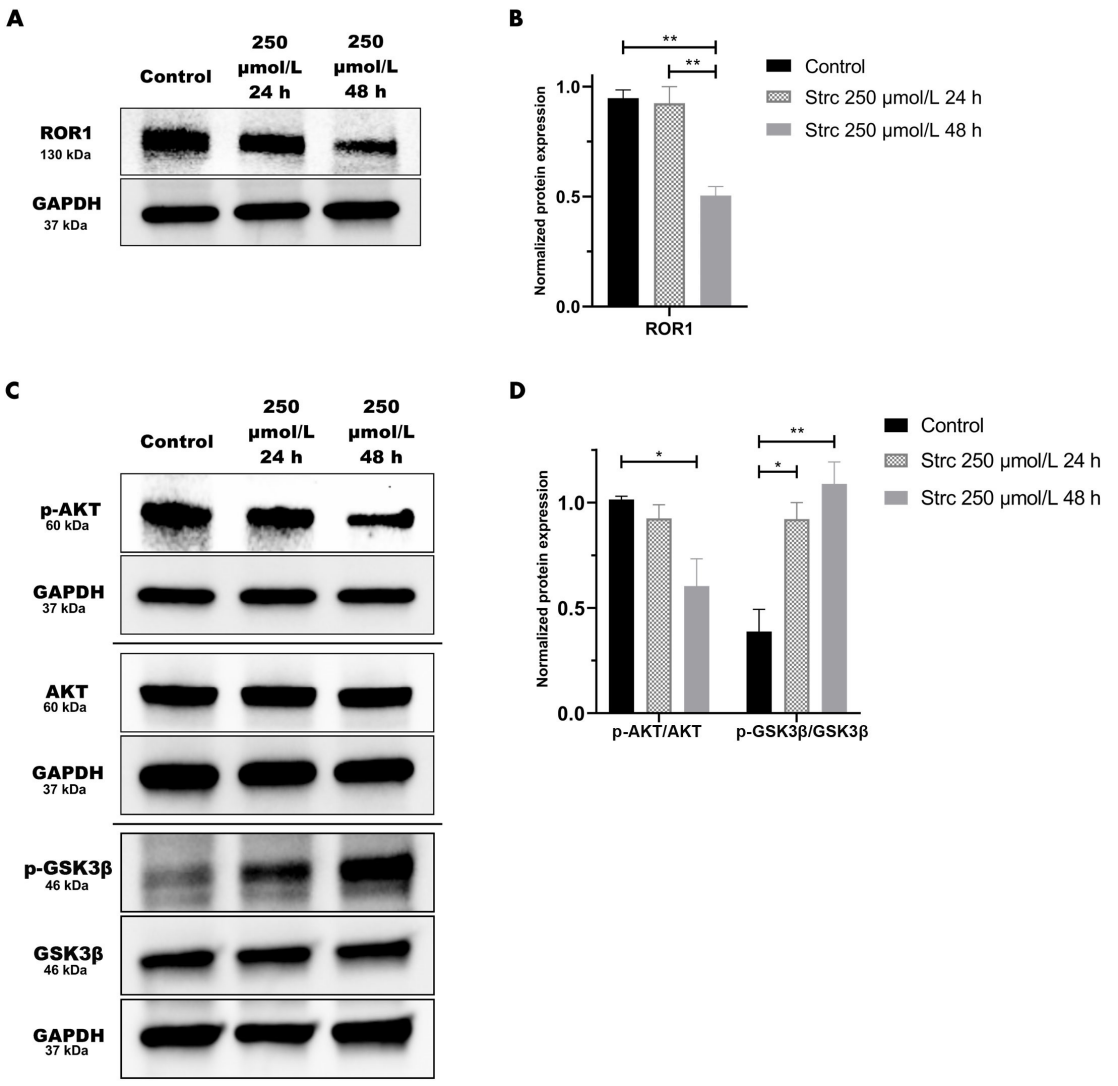
Strictinin modulates ROR1-mediated AKT-GSK3β signaling in DU145 cells. (A) Immunoblot on DU145 cells treated with control, 250 µmol/L strictinin for 24 h, or 250 µmol/L strictinin for 48 h. ROR1 is visualized; (B) quantification of ROR1 protein band normalized to GAPDH. Data are represented as the mean ± SEM from three independent western blot experiments; (C) immunoblot on DU145 cells treated with control, 250 µmol/L strictinin for 24 h, or 250 µmol/L strictinin for 48 h. AKT, p-AKT-ser473, GSK3β, p-GSK3β-ser9 visualized; (D) quantification of p-AKT/AKT and p-GSK3β/GSK3β protein bands normalized to GAPDH. Data are represented as the mean ± SEM from three independent western blot experiments. *N* = 3. ^**^ *P* value < 0.01; ^*^ *P* value < 0.05

Strictinin impacted ROR1 protein expression and downstream oncogenic signaling in PC3 cells (Figure 3). Hence, we wanted to determine whether strictinin inhibited ROR1 and modulated the AKT-GSK3β pathway in DU145 cells that also express ROR1 [30]. The concentrations of strictinin that were used to treat PC3 cells were also used to treat DU145 cells in order to evaluate how the same concentrations of strictinin impacted PC3 and DU145 cell signaling cascades. We found that the protein expression of ROR1 and p-AKT-ser473 was diminished, especially in the 48 h strictinin treatment condition in comparison to the control (Figure 8A–D). Interestingly, we identified that DU145 cells treated with strictinin had increased levels of p-GSK3β-ser9 in comparison to the control (Figure 8C and D). These results indicate that strictinin mitigates the expression of ROR1 in DU145 cells, which leads to the inhibition of downstream p-AKT (Figure 2C). However, strictinin increases the phosphorylation of GSK3β in DU145 cells, perhaps by indirectly influencing GSK3β outside of the ROR1 signaling axis (Figure 2C).

### Strictinin exhibits anti-cancer effects in DU145 cells

**Figure 9:**
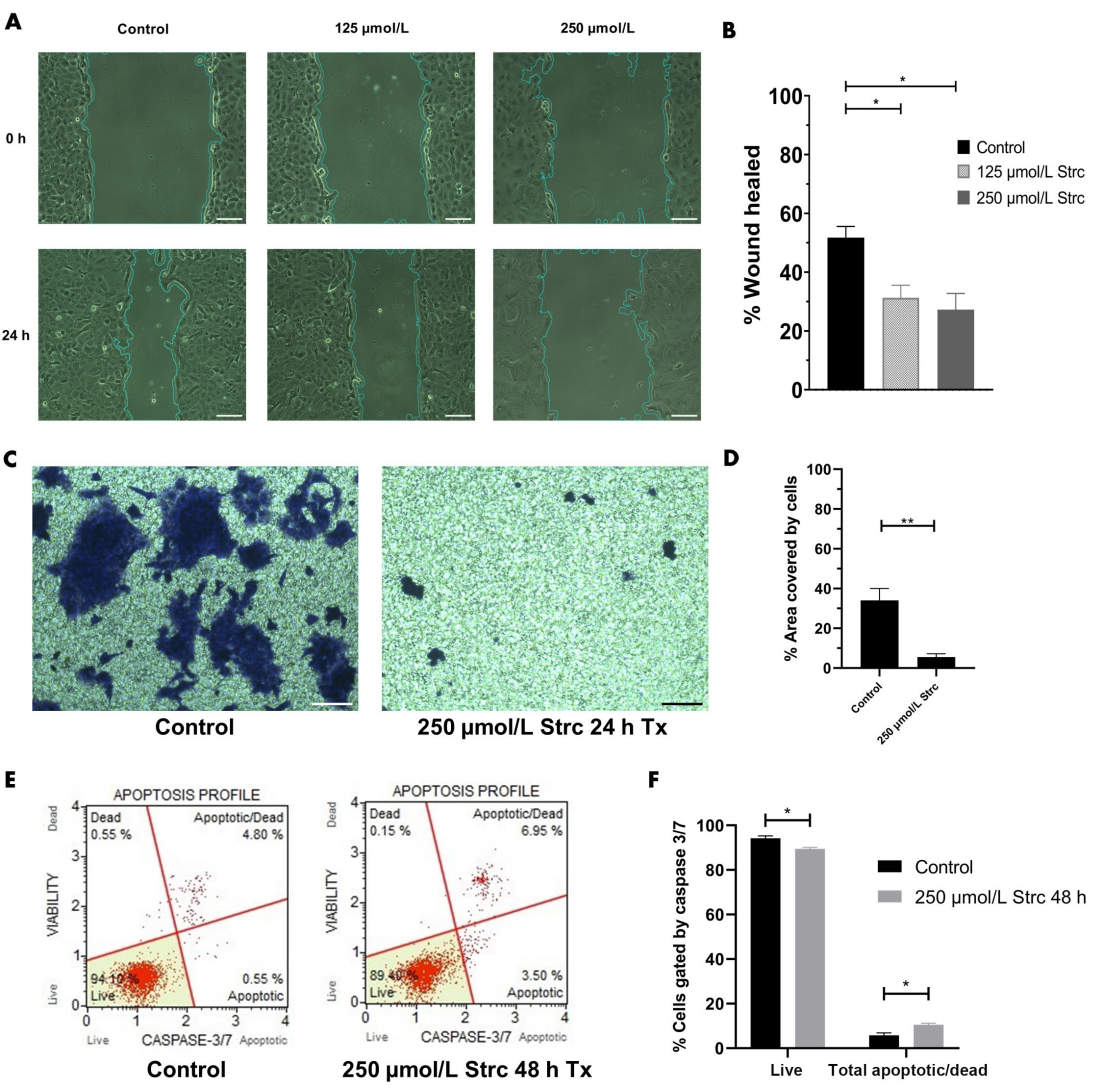
Strictinin exhibits anti-migratory, anti-invasive, and minimal apoptotic effects on DU145 cells. (A) Wound healing assay of DU145 cells treated with control, 125 µmol/L strictinin, or 250 µmol/L strictinin for 24 h. All images were taken at 10× magnification. Scale bar represents 200 pixels; (B) wound healing data quantified and represented as percentage wound healed to assess the migratory capacity of DU145 cells under different treatment conditions; (C) bright field image of invasive, crystal-violet stained DU145 cells after treatment with control or 250 µmol/L strictinin for 24 h. All images were taken at 10× magnification. Scale bar represents 200 pixels; (D) invasion of the cells were quantified as percentage area covered by the cells; (E) Muse caspase 3/7 flow cytometry of DU145 cells treated with control or strictinin. Cells were categorized into live, apoptotic, apoptotic/dead, or dead based on fluorescence intensity of caspase 3 and caspase 7; (F) quantification of caspase 3/7 flow cytometry on treated DU145 cells. *N* ≥ 3. ^**^ *P* value < 0.01; ^*^ *P* value < 0.05

Our previous research identified that DU145 cells have a moderate level of ROR1 protein expression that is greater than RWPE-1 cells but less than PC3 cells [30]. Hence, we sought to identify whether strictinin impacted DU145 cell migration, invasion, and apoptosis. The concentrations of strictinin that were used to treat PC3 cells were also used to treat DU145 cells in order to compare the effectiveness of strictinin in DU145 *versus* PC3. DU145 cell migration and invasion were inhibited upon treatment with strictinin (Figure 9A–D). However, strictinin minimally impacted pro-apoptotic caspase 3/7 expression and did not substantially affect apoptosis of DU145 cells (Figure 9E and F). Taken together, the data suggests that strictinin can exhibit anti-cancerous effects against DU145 cells but is less lethal to DU145 in comparison to PC3 cells. This is most likely due to the lower levels of ROR1 expression that DU145 cells display in comparison to PC3 cells. Strictinin likely represses the migration and invasion of DU145 cells through its ability to modulate ROR1-mediated AKT-GSK3β signaling (Figure 8 and Figure 2C).

## Discussion

Prostate cancer, though initially susceptible to hormone therapy, can progress through mechanisms that cause androgen signaling to thrive despite low levels of circulating androgen, or by upregulating oncogenic signaling axes that are completely independent of androgen [12, 50, 51]. Hormone therapies like androgen receptor antagonists (e.g., enzalutamide) and androgen synthesis inhibitors (e.g., abiraterone) are increasing the occurrence of a subtype of prostate cancer that is negative for the androgen receptor and can progress via AI oncogenic signaling cascades [12–14]. This subtype of prostate cancer, which can be classified as AR^neg^-AI, is difficult to treat. Despite progress in prostate cancer therapies, the mean survival of patients with CRPC is about 3 years at maximum [52]. Therefore, we must explore tumor-associated targets and therapies that are more specific to aggressive prostate cancer.

ROR1 is an oncofetal RTK-like protein that has an emerging role in cancer. The protein is normally expressed during embryonic development but exhibits minimal to no expression in fully developed tissue [15–17]. In cancer, ROR1 is highly dysregulated and plays a key role in tumor progression [35, 36, 53, 54]. Previous studies have shown that inhibiting ROR1 effectively mitigates aggressive cancer phenotypes [23–25]. Our lab has further identified that inhibiting ROR1 suppresses TNBC survival. In our previous study by Fultang et al. [29], it was revealed that the natural compound strictinin bound to ROR1 and competitively inhibited the binding of Wnt5a [29]. This suppressed the downstream PI3K-AKT-GSK3β pathway, which led to the inhibition of migration, invasion, and anti-apoptosis in TNBC [29].

Since ROR1 expression is upregulated in prostate cancer, we hypothesized that strictinin could target aggressive prostate cancer. This study identifies strictinin as an effective anti-cancer compound that significantly targets AR^neg^-AI PC3 cells, moderately targets AR^neg^-AI DU145 cells, and minimally impacts the functions of normal prostate epithelium RWPE-1. Strictinin suppresses ROR1 expression and modulates downstream signaling cascades that play a role in anti-apoptosis, migration, invasion, and EMT, which mitigates prostate cancer cell aggressiveness. The inhibition of ROR1 expression by strictinin likely means that endogenous ligands (e.g., Wnt5a) would not be able to effectively activate the receptor and downstream oncogenic signaling.

First, we wanted to evaluate the effects of strictinin on PC3 and RWPE-1 cell viability and apoptosis. Since we previously characterized strictinin as an ROR1 inhibitor, we posited that strictinin would be selectively lethal to ROR1-expressing PC3 cells in comparison to RWPE-1 cells. Indeed, our MTT, Annexin V fluorescence, and apoptosis flow cytometry results demonstrated that strictinin was significantly more cytotoxic to PC3 cells and induced apoptosis of PC3 in comparison to RWPE-1 cells (Figure 1). The 72 h MTT proliferation studies revealed that the IC_50_ of strictinin in PC3 cells (277.2 µmol/L) was approximately 2-fold lower in comparison to RWPE-1 cells (658.6 µmol/L) (Figure 1A). Furthermore, treatment of RWPE-1 cells with 250 µmol/L strictinin for 72 h did not significantly increase Annexin V phosphatidylserine expression in comparison to the control treatment (Figure 1B). Since strictinin did not seem to significantly increase apoptosis of RWPE-1 cells, we wanted to evaluate apoptosis in PC3. Flow cytometry results revealed that the expression of Annexin V, 7-AAD, pro-apoptotic caspase 3, and pro-apoptotic caspase 7 significantly increased in PC3 cells treated with 250 µmol/L strictinin for 48 h to 72 h in comparison to the control (Figure 1C–E). As a result, the flow cytometry data also demonstrated that strictinin-treated PC3 cells underwent significant apoptosis (Figure 1D and E, Figure S1).

We predicted that the strictinin-induced cytotoxicity and apoptosis in PC3 cells was due to its ability to inhibit ROR1-mediated oncogenic signaling. Hence, we wanted to determine the molecular mechanism by which strictinin was exhibiting its anti-cancer effect on PC3 cells. Strictinin was found to suppress the expression of ROR1 in PC3 cells, which led to a reduction in the PI3K-AKT-GSK3β signaling cascade (Figure 3). This data suggests that strictinin can exhibit lethality against PC3 cells by targeting ROR1 and inhibiting PI3K-AKT-GSK3β signaling, which is a key pathway that drives many of the malignant properties of cancer. In particular, p-AKT can increase the activity of anti-apoptotic proteins p-B-cell lymphoma 2 (BCL2)-associated agonist of cell death (Bad) and X-linked inhibitor of apoptosis (XIAP), which leads to the prevention of apoptosis (Figure 2A) [29]. Prior studies in our lab demonstrated that strictinin inhibited the expression of p-AKT, p-Bad (ser136), and XIAP in TNBC cells [29]. Our results suggest that the reduction in p-AKT caused by strictinin most likely mitigates the functions of anti-apoptotic proteins and leads to the significant apoptosis of PC3 cells, as supported by the increased expression of Annexin V, 7-AAD, caspase 3, and caspase 7 seen in PC3 cells treated with strictinin (Figure 1C–E, Figure S1, Figure 2B).

ROR1 and the downstream PI3K-AKT-GSK3β pathway are important drivers of cancerous phenotypes, such as aggressive metastasis that is driven by increased cell migration and invasion [29, 34–36, 55]. Therefore, we wanted to evaluate the effects of strictinin on PC3 cell migration and invasion. We found that strictinin markedly suppressed the migration and invasion of PC3 cells while having a minimal impact on RWPE-1 cell phenotype (Figure 4, Figure 5A–C). To understand the underlying molecular phenomenon behind this change, we evaluated the expression of EMT markers Twist1, Snail, and MMP9, which are known to be upregulated in the wake of increased PI3K-AKT activity (Figure 2A) [37–40]. EMT is an important process in cancer that allows cancer cells to lose cell-to-cell adhesion and polarity while gaining mesenchymal characteristics that increase migratory and invasive potential [37–40]. We identified that strictinin negatively impacted the expression of Twist1, Snail, and MMP9 in PC3 cells (Figure 5D and E). This finding implies that strictinin can repress migration and invasion of aggressive PC3 cells by diminishing the expression of MMP9, Twist1, and Snail (Figure 2B). The reduction in EMT markers also suggests that strictinin may induce MET, a phenomenon that most likely underscores the reduced migratory and invasive capacity of strictinin-treated PC3 cells.

ROR1 and the PI3K-AKT pathway also affect cell cycle dynamics [56–58]. Hence, we wanted to investigate the effects of strictinin on the PC3 and RWPE-1 cell cycle. Strictinin was found to generate S-phase cell cycle arrest in both PC3 and RWPE-1 cells (Figure 6). However, strictinin caused a greater magnitude of S-phase cell cycle arrest of PC3 cells in comparison to RWPE-1 (~5% *vs.* ~2%), which indicates that strictinin more selectively impacts PC3 cell cycle dynamics (Figure 6B and D). The PI3K-AKT pathway is known to increase the expression of cyclin D1 and cyclin A2, which allow for S-phase progression and G2/M-phase progression, respectively (Figure 2A) [41–44, 56]. Thus, the inhibition of ROR1 and the PI3K-AKT-GSK3β pathway by strictinin could negatively impact cyclin D1 and cyclin A2 expression in PC3 cells (Figure 2B). Exploring the expression of cyclin proteins in PC3 cells treated with strictinin could further unravel the complex molecular pathway by which strictinin exerts its anti-cancerous effects in relation to the cell cycle.

To strengthen our findings on the targeted and inhibitory effects that strictinin has on ROR1-expressing prostate cancer cells, we wanted to evaluate its potential synergism with docetaxel, which is a standard chemotherapeutic agent that is provided at 75 mg/m^2^ intravenously (IV) for 1 h every 3 weeks in patients with metastatic prostate cancer [46, 59, 60]. Docetaxel exerts its antineoplastic effects by preventing microtubule depolymerization and reducing the expression of anti-apoptotic gene B-cell lymphoma 2 (*BCL2*) [61]. The reported IC_50_ of docetaxel in PC3 cells varies substantially in the literature [62–65]. Therefore, we treated PC3 cells with docetaxel alone and obtained an IC_50_ of 452.7 nmol/L, which we were able to use as a baseline for comparison when performing combination therapy experiments (Figure S3B). We discovered that a combination of strictinin and docetaxel treatment in PC3 cells exhibited strong synergism as shown by the isobologram and a CI of 0.311 (Figure 7B). In aggressive PC3 cells, combination strictinin and docetaxel significantly reduced the IC_50_ of strictinin by about 7-fold (277.2 µmol/L to 38.71 µmol/L) and docetaxel by about 6-fold (452.7 nmol/L to 77.43 nmol/L) when compared to the IC_50_ of strictinin and docetaxel used alone (Figure S3A and B). Though the IC_50_ of strictinin in PC3 cells (277.2 µmol/L) was approximately 2-fold lower in comparison to RWPE-1 cells, the IC_50_ is quite high and comparable to our previous study in TNBC (MDA-MB-231) [29]. The combination treatment significantly lowering the IC_50_ of strictinin in PC3 cells means that strictinin can be provided at a much lower dose when combined with docetaxel. In general, chemotherapy can often result in side effects that can lead to chemotherapy cessation and negatively impact patient outcomes [46, 48, 49, 66]. The synergism between strictinin and docetaxel *in vitro* may imply that the 75 mg/m^2^ IV dose of docetaxel can be lowered along with strictinin to achieve the desired therapeutic effect in patients. Utilizing less docetaxel in combination with strictinin may help alleviate harsh chemotherapy side effects in prostate cancer patients while enabling more specificity of therapy by strictinin-induced targeting of ROR1. This may aid in overall survival and improve patient outcomes in prostate cancer therapy.

Since we evaluated how strictinin affected ROR1-mediated AKT-GSK3β signaling in PC3 cells, we also wanted to determine the impact of strictinin on ROR1 and AKT-GSK3β signaling in DU145, which is another AR^neg^-AI, ROR1 expressing prostate cancer cell line [30]. We found that although strictinin inhibited the expression of ROR1 and p-AKT-ser473, strictinin increased the expression of p-GSK3β-ser9 in DU145 cells (Figure 8). Strictinin suppresses the protein expression of ROR1 and p-AKT in DU145 cells but increases the phosphorylation of GSK3β, possibly by indirectly acting on GSK3β outside of the ROR1 signaling pathway (Figure 2C). Finally, we wanted to evaluate the phenotypic effects of strictinin on DU145 cells. However, because our prior study revealed that ROR1 expression was lower in DU145 cells in comparison to PC3 cells, we hypothesized that strictinin would suppress DU145, but to a lesser extent than PC3 [30]. Indeed, our data demonstrated that strictinin mitigated DU145 cell migration and invasion, while minimally impacting apoptosis (Figure 9). It is possible that DU145 cells rely less on ROR1-AKT signaling since DU145 cells express lower levels of ROR1 and p-AKT-ser473 in comparison to PC3 cells [30, 67–69]. Therefore, the inhibition of ROR1-AKT signaling by strictinin may not lead to significant apoptosis, but may contribute to the reduction of migration and invasion in DU145 cells (Figures 8 and 9) [70, 71]. These findings suggest that strictinin is still an effective compound for treating prostate cancers that exhibit molecular and phenotypic characteristics like DU145, especially if the goal is to regulate metastasis. However, combination therapy of strictinin and docetaxel may have to be utilized in DU145-like prostate cancers if the goal is to induce significant cytotoxicity or apoptosis. Since AR^neg^-AI prostate cancers lack cancer-associated markers, ROR1 inhibition by strictinin may still be a suitable option for DU145-like clinical prostate cancer.

GSK3β can have both pro-cancerous and anti-cancerous effects, which may explain the difference in the phosphorylation of GSK3β observed in strictinin-treated PC3 and DU145 cells [72, 73]. The reduced phosphorylation of GSK3β allows the activated protein to decrease the expression of Snail [39, 73]. This phenomenon can lead to anti-migratory and anti-invasive effects as demonstrated by strictinin-treated PC3 cells (Figure 2B, Figure 3C and D, Figure 4A and B, Figure 5A, B, D, and E) [39, 73]. Though the inhibition of GSK3β (increase in p-GSK3β-ser9) is a common feature seen in many cancers, inhibiting GSK3β activity can also be tumor-suppressive and can reduce the migration and invasion of cancers [72–74]. In strictinin-treated DU145 cells, we may be observing that the increase in p-GSK3β suppresses migration and invasion (Figure 2C, Figure 8C and D, Figure 9A–D) [74]. GSK3β most likely functions as a tumor suppressor in PC3 and as a tumor promoter in DU145, which may explain why strictinin decreases the phosphorylation of GSK3β (active) in PC3 cells and increases the phosphorylation of GSK3β (inactive) in DU145 cells [72–74]. The reduction of p-GSK3β-ser9 in PC3 cells treated with strictinin and the increase of p-GSK3β-ser9 in DU145 cells treated with strictinin likely leads to the anti-migratory and anti-invasive effects that are observed in both cell lines (Figure 2B and C).

In this study, we establish strictinin as an effective, natural small-molecule inhibitor that can suppress highly aggressive AR^neg^-AI prostate cancer. Though we identify that strictinin induces anti-cancer effects via the modulation of ROR1-mediated AKT-GSK3β signaling, it is important to note that strictinin may have other modes of action that warrant further investigation. Our findings suggest that strictinin can selectively target prostate cancers that express ROR1 to hinder anti-apoptosis, migration, invasion, EMT, and cell cycle progression. By identifying the molecular mechanism by which strictinin negatively impacts prostate cancer, we highlight the importance of ROR1 as a driver of aggressive prostate cancer. Furthermore, strictinin is synergistic with docetaxel in PC3 cells, which may enable the utilization of combination therapy to boost the specificity of treatment while limiting the harmful effects of chemotherapy. Our study reveals that strictinin may have marked utility for the treatment of AR^neg^-AI prostate cancer and that ROR1 is an effective marker for targeted prostate cancer treatment.

ROR1 plays an important role in a variety of malignancies. A study found that ROR1 expression was upregulated in pancreatic cancer tissue in comparison to adjacent noncancerous tissue [35]. The study also identified that knocking down ROR1 inhibited EMT and invasion of circulating tumor cells that were obtained from blood samples of patients who had pancreatic cancer [35]. Daneshmanesh et al. [23] discovered that a small molecule inhibitor of ROR1 (KAN0439834) caused the apoptosis of pancreatic cancer cells. ROR1 is also an important biomarker in lung adenocarcinoma. A study found that ROR1 expression was increased in lung adenocarcinoma in comparison to noncancerous lung tissue and that high ROR1 expression was associated with poor patient prognosis [54]. Furthermore, Liu et al. [25] found that knocking down ROR1 suppressed proliferation and induced apoptosis in non-small cell lung cancer cells. The importance of ROR1 has also been characterized in chronic lymphocytic leukemia, diffuse large B-cell lymphoma, melanoma, ovarian cancer, and breast cancer [24, 36, 53, 75, 76]. Our lab (Fultang et al. [29]) has previously investigated the anti-proliferative, anti-migratory, anti-invasive, and pro-apoptotic effects of ROR1 inhibition by strictinin in TNBC. It is worth investigating strictinin as a ROR1 inhibitor in other cancers that display elevated levels of ROR1 expression to further expand on the findings in this study that identify the anti-cancerous effects of strictinin in AR^neg^-AI prostate cancer.

## Abbreviations

7-AAD: 7-aminoactinomycin D
AI: androgen-independent
AKT: protein kinase B
AR^neg^: androgen-receptor negative
ATCC: American Type Culture Collection
CI: combination index
CRPC: castration-resistant prostate cancer
DMSO: dimethyl sulfoxide
EMT: epithelial-to-mesenchymal transition
FBS: fetal bovine serum
GAPDH: glyceraldehyde-3-phosphate dehydrogenase
GSK3β: glycogen synthase kinase 3beta
IC_50_: half-maximal drug inhibitory concentration
MMP9: matrix metalloproteinase 9
MTT: methyl thiazolyl tetrazolium
PBS: phosphate buffer solution
PI3K: phosphatidylinositol 3-kinase
ROR1: receptor tyrosine kinase-like orphan receptor 1
RTK: receptor tyrosine kinase
SEM: standard error of the mean
TNBC: triple-negative breast cancer

## Supplementary materials

The supplementary Figures for this article are available at: https://www.explorationpub.com/uploads/Article/file/1002192_sup_1.pdf. Other supplementary material for this article is available at: https://www.explorationpub.com/uploads/Article/file/1002192_sup_2.pdf.

## Declarations

### Availability of data and materials

All datasets generated or analyzed for this study are included in the manuscript and the supplementary files.

### Copyright

© The Author(s) 2023.
