# Supplementary material for: Receptor tyrosine kinase-like orphan receptor 1 inhibitor strictinin exhibits anti-cancer properties against highly aggressive androgen-independent prostate cancer
Source: Explor Target Antitumor Ther. 2023 Dec 20;4(6):1188–209. doi: 10.37349/etat.2023.00192 (PMC10784114; doi:10.37349/etat.2023.00192)
Supplement: Supplementary file 1 [file 1002192_sup_1.pdf]

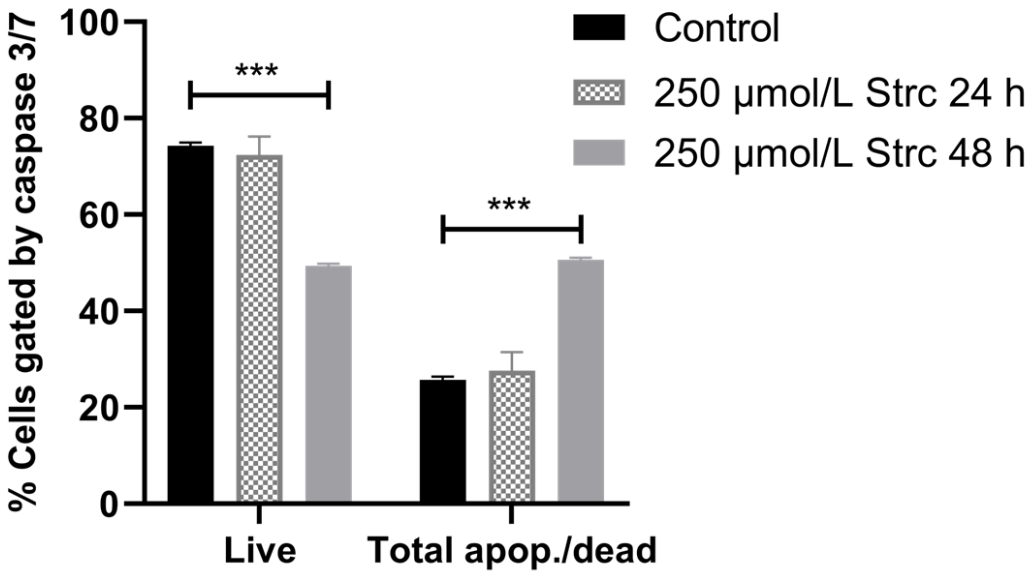

Figure S1. Strictinin causes the apoptosis of PC3 cells. Quantification of caspase 3/7 flow cytometry on treated PC3 cells. Cells were categorized into live, apoptotic, apoptotic/dead, or dead based on fluorescence intensity of caspase 3 and caspase 7. $N = 3$. *** P value < 0.001. apop: apoptosis

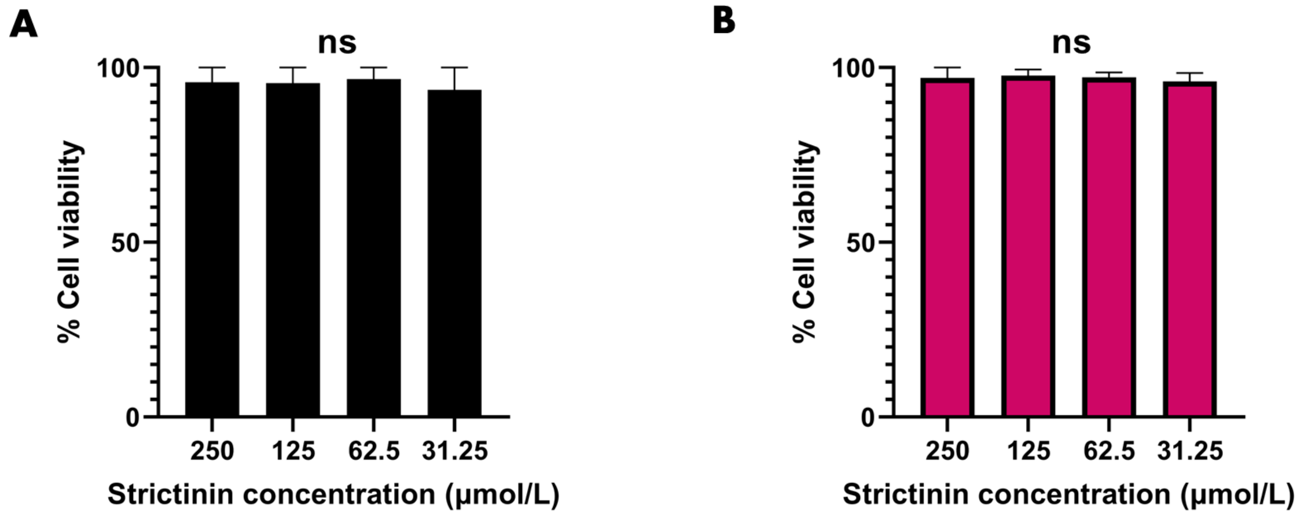

Figure S2. Strictinin treatment at 24 hours does not impact the cell viability of PC3 and RWPE-1. (A) Cell viability of PC3 after treatment with different strictinin concentration for 24 hours. (B) cell viability of RWPE-1 after treatment with different strictinin concentrations for 24 hours. $N = 3$. ns: not significant

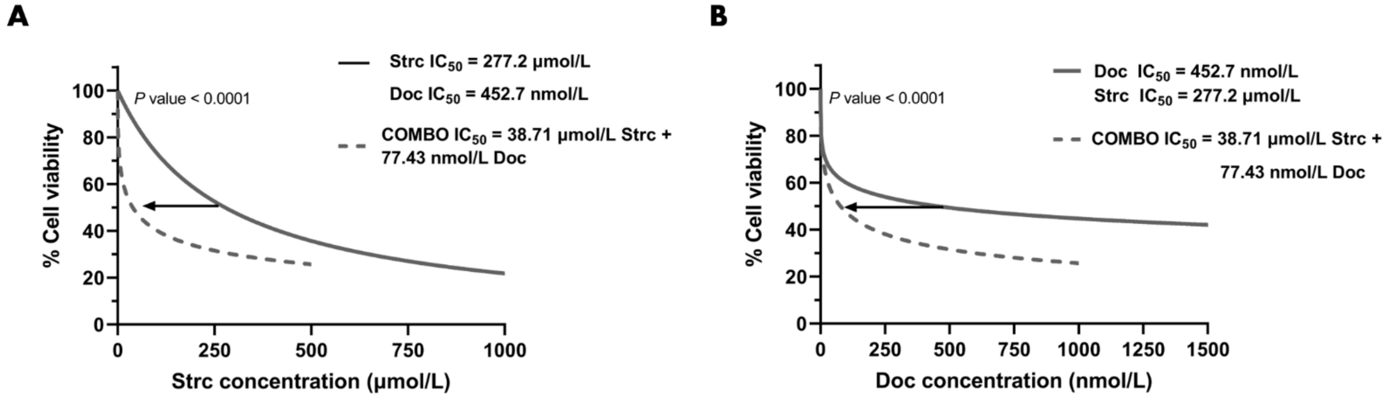

Figure S3. Combination strictinin and docetaxel treatment exhibits synergism in PC3 cells. (A) Cell viability of PC3 after combination strictinin and docetaxel treatment. Data are plotted against strictinin concentration. (B) cell viability of PC3 after combination strictinin and docetaxel treatment. Data are plotted against docetaxel concentration. $N \geq 3$
