# Supplementary material for: Receptor tyrosine kinase-like orphan receptor 1 inhibitor strictinin exhibits anti-cancer properties against highly aggressive androgen-independent prostate cancer
Source: Explor Target Antitumor Ther. 2023 Dec 20;4(6):1188–209. doi: 10.37349/etat.2023.00192 (PMC10784114; doi:10.37349/etat.2023.00192)
Supplement: Supplementary file 2 [file 1002192_sup_2.pdf]

Raw western blots (Figures 1-8)

1. All lanes used in the manuscript and used for densitometry analysis (N=3) are labeled. Unlabeled lanes were not used for analysis.
2. All proteins used in the manuscript and used for analysis are appropriately labeled. Unlabeled proteins were either not used for analysis or were labeled in subsequent slides.
3. All protein lysate was obtained from PC3 cells.
4. All densitometry analysis was normalized to housekeeping protein (GAPDH).
5. Blue lines indicates how the blot was spliced for final presentation.
6. Single blue line indicates where the blot was physically cut.
7. Red boxes indicate how the blot was cropped for publication (approximately).
8. Labeling:
   - Lanes
     - 1: Control
     - 2: Strc 250 μM- 24h treatment
     - 3: Strc 250 μM- 48h treatment
   - Proteins
     - pGSK3b = phospho-GSK3β (ser9)
     - pAKT = phospho-AKT (ser473)

Figure 1

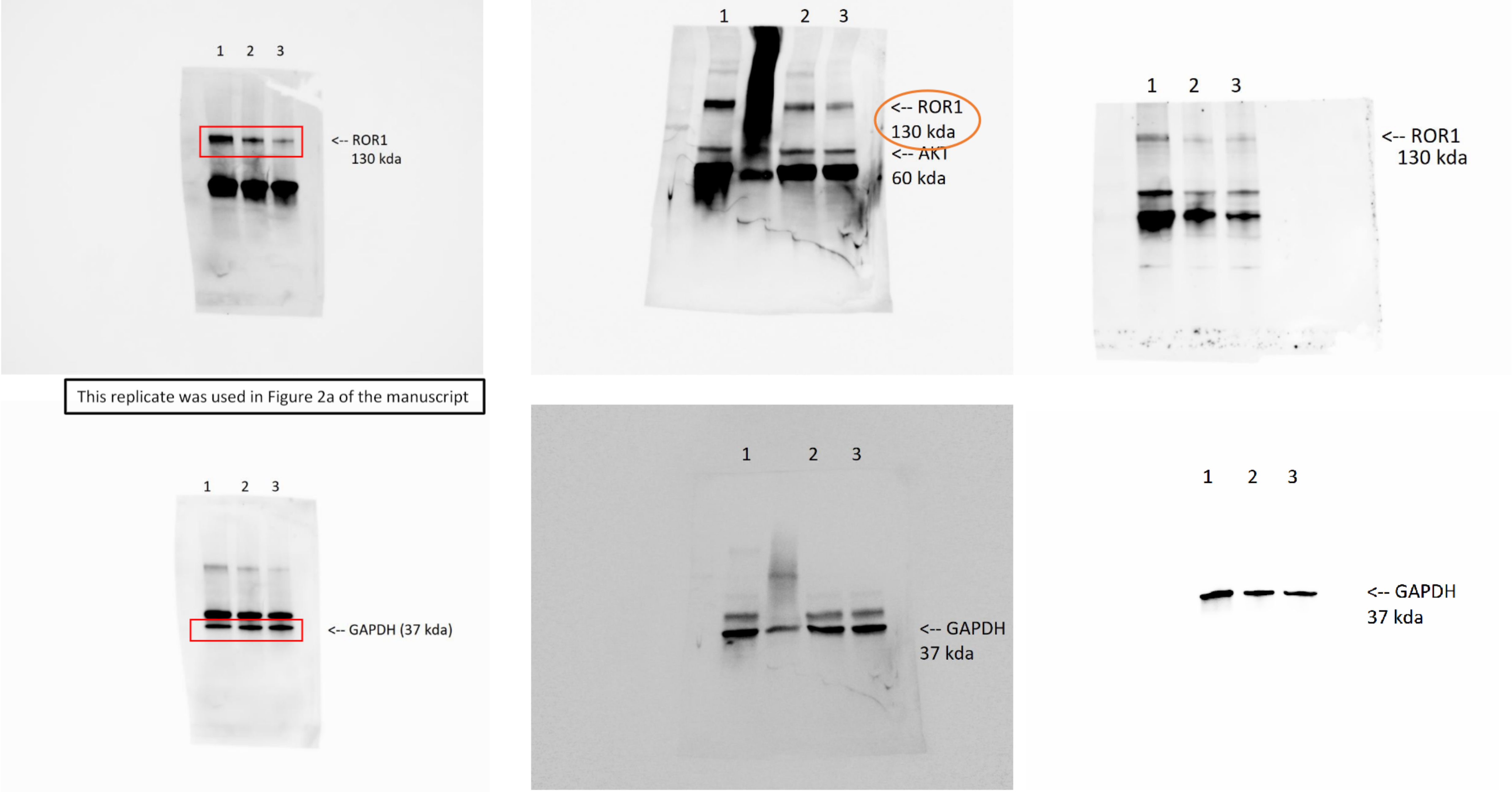
1 2 3
<-- ROR1 130 kda
1 2 3
<-- ROR1 130 kda
<-- AKT 60 kda
1 2 3
<-- ROR1 130 kda
This replicate was used in Figure 2a of the manuscript
1 2 3
<-- GAPDH (37 kda)
1 2 3
<-- GAPDH 37 kda
1 2 3
<-- GAPDH 37 kda

Figure 2

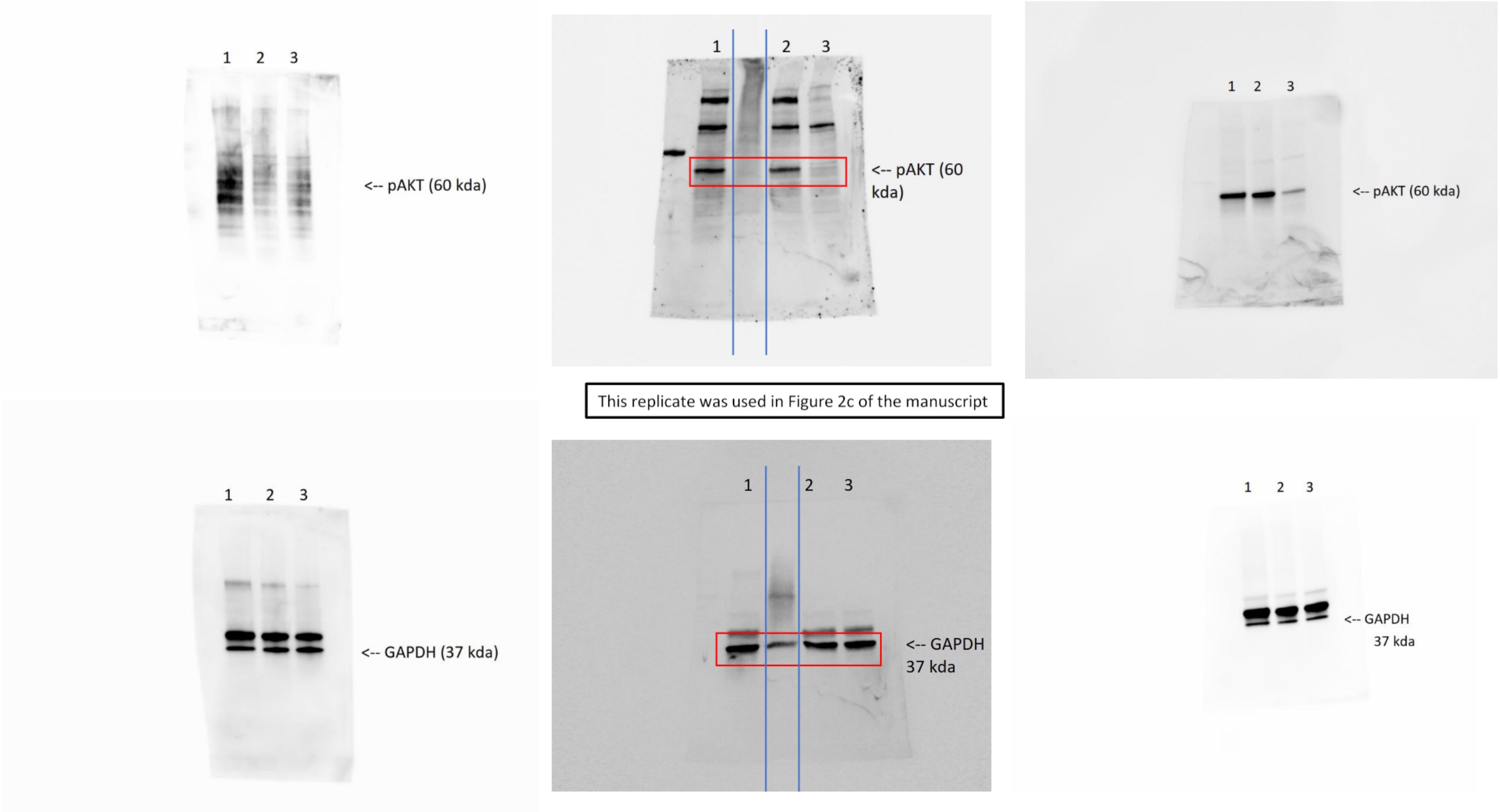

Vertical blue lines indicate how the blot was spliced for final presentation

Figure 3

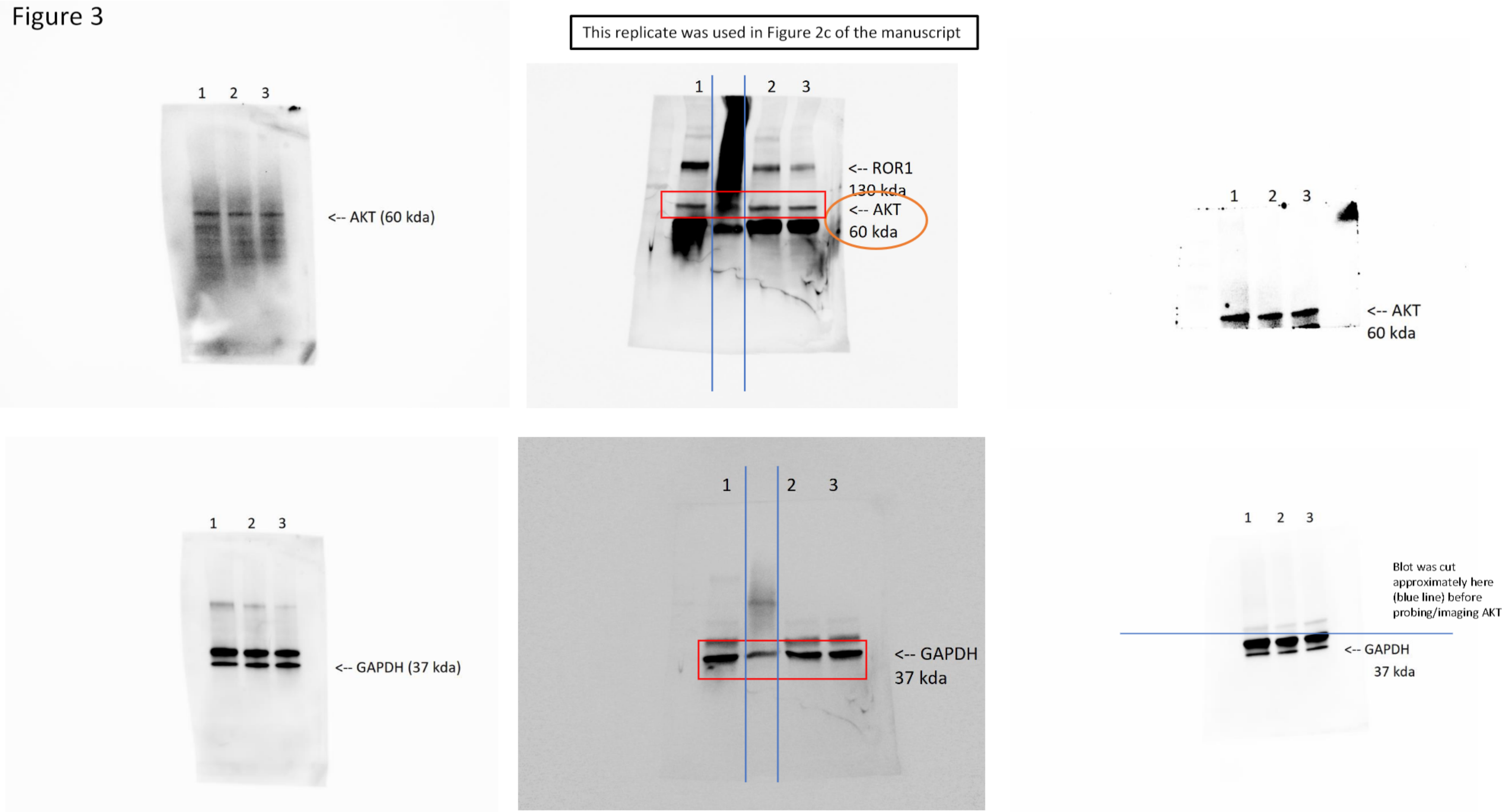

Vertical blue lines indicate how the blot was spliced for final presentation

Figure 4

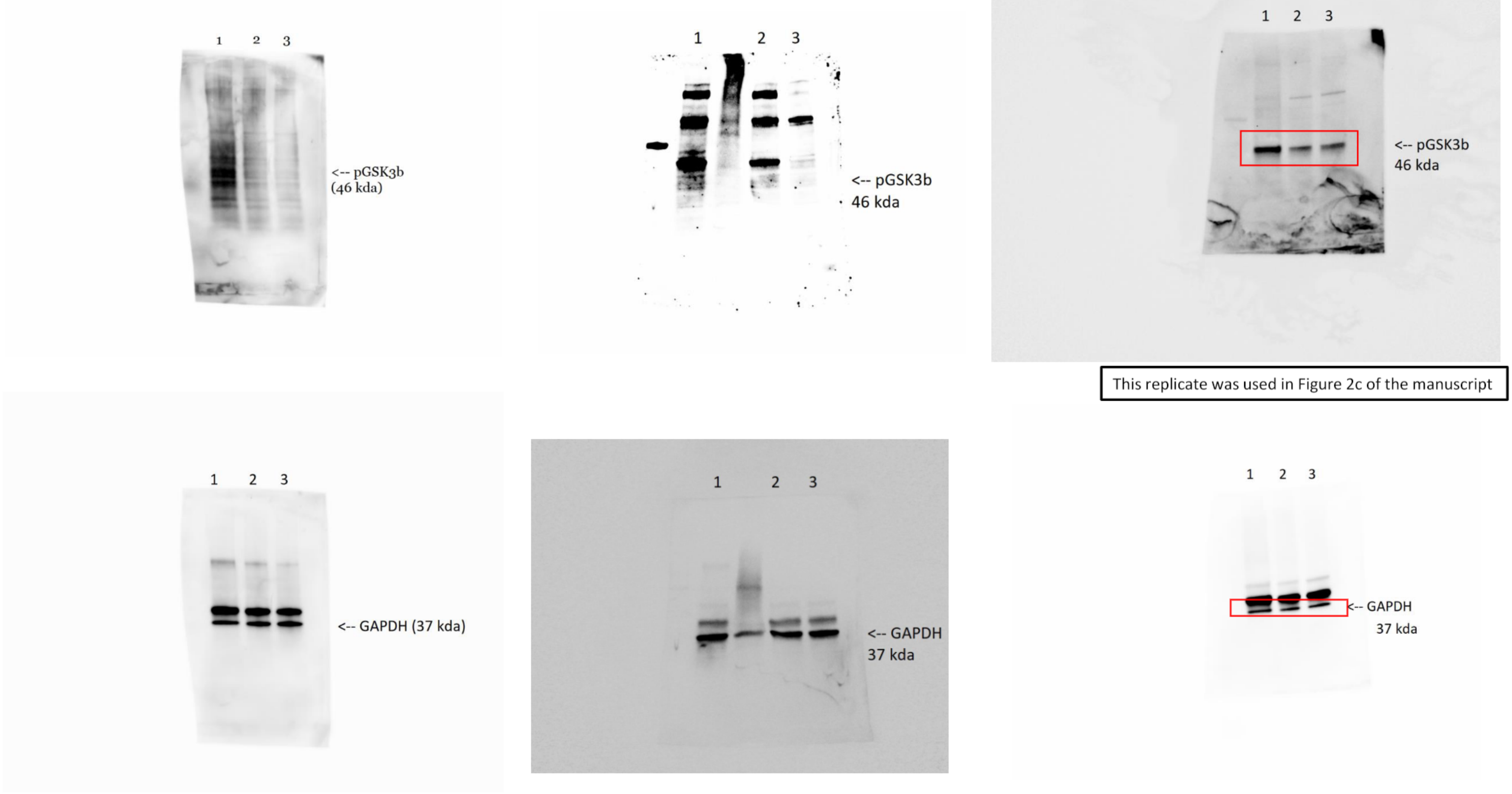
1 2 3
<-- pGSK3b
(46 kda)
1 2 3
<-- pGSK3b
46 kda
1 2 3
<-- pGSK3b
46 kda
This replicate was used in Figure 2c of the manuscript
1 2 3
<-- GAPDH (37 kda)
1 2 3
<-- GAPDH
37 kda
1 2 3
<-- GAPDH
37 kda

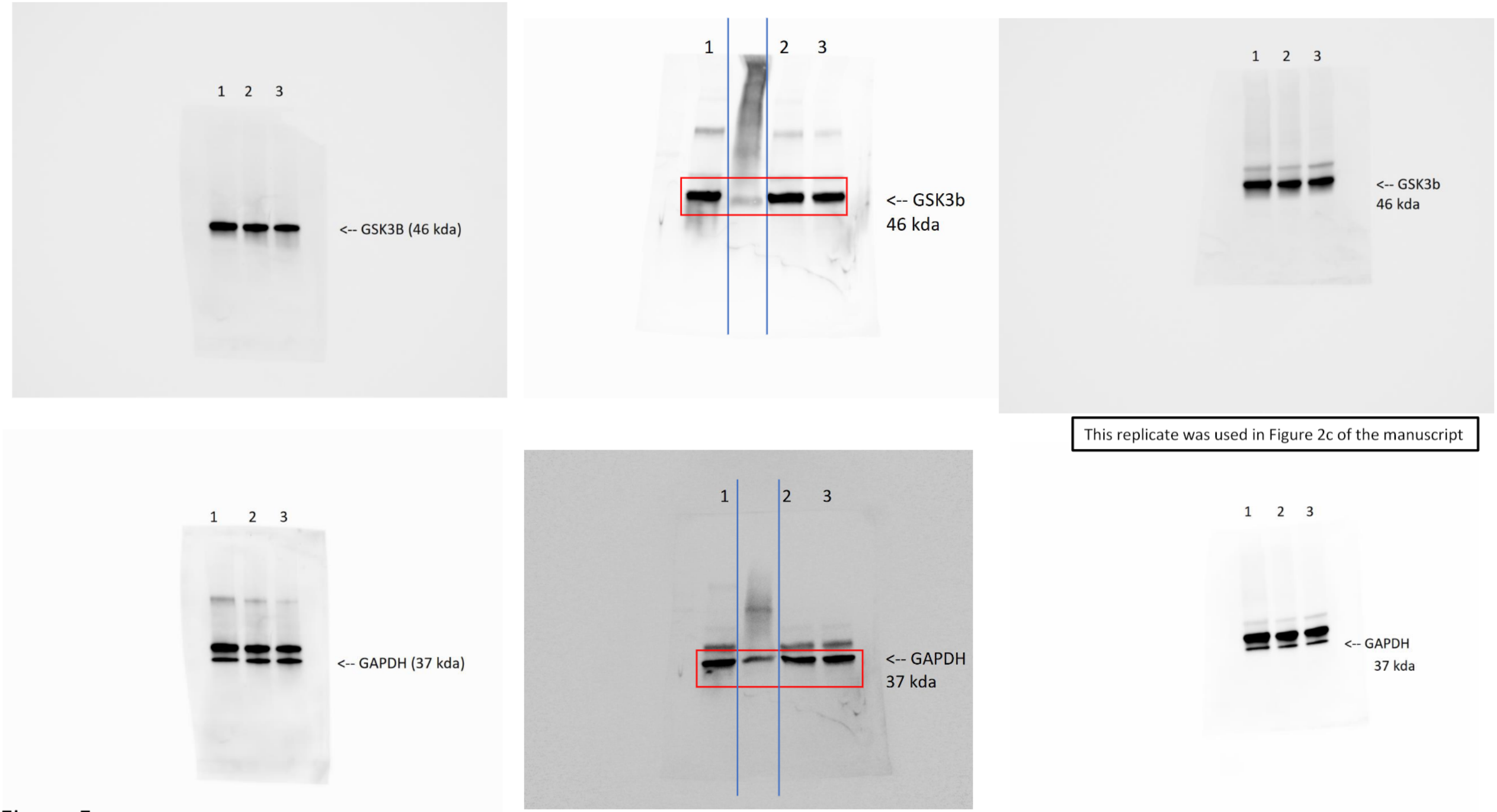

Figure 5

Vertical blue lines indicate how the blot was spliced for final presentation

Figure 6

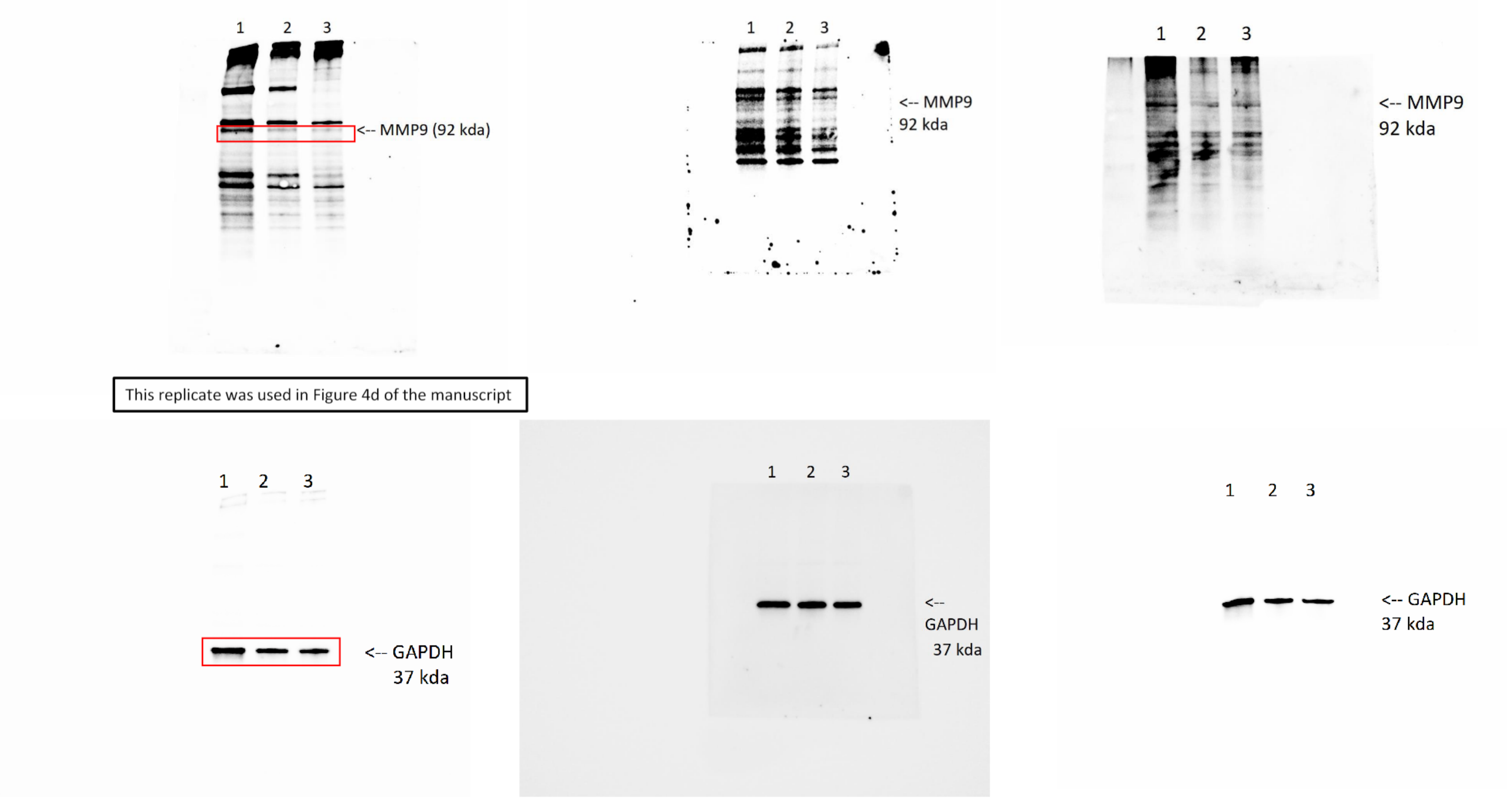

1 2 3
<-- MMP9 (92 kda)
1 2 3
<-- MMP9
92 kda
1 2 3
<-- MMP9
92 kda
This replicate was used in Figure 4d of the manuscript
1 2 3
<-- GAPDH
37 kda
1 2 3
<--
GAPDH
37 kda
1 2 3
<-- GAPDH
37 kda

Figure 7

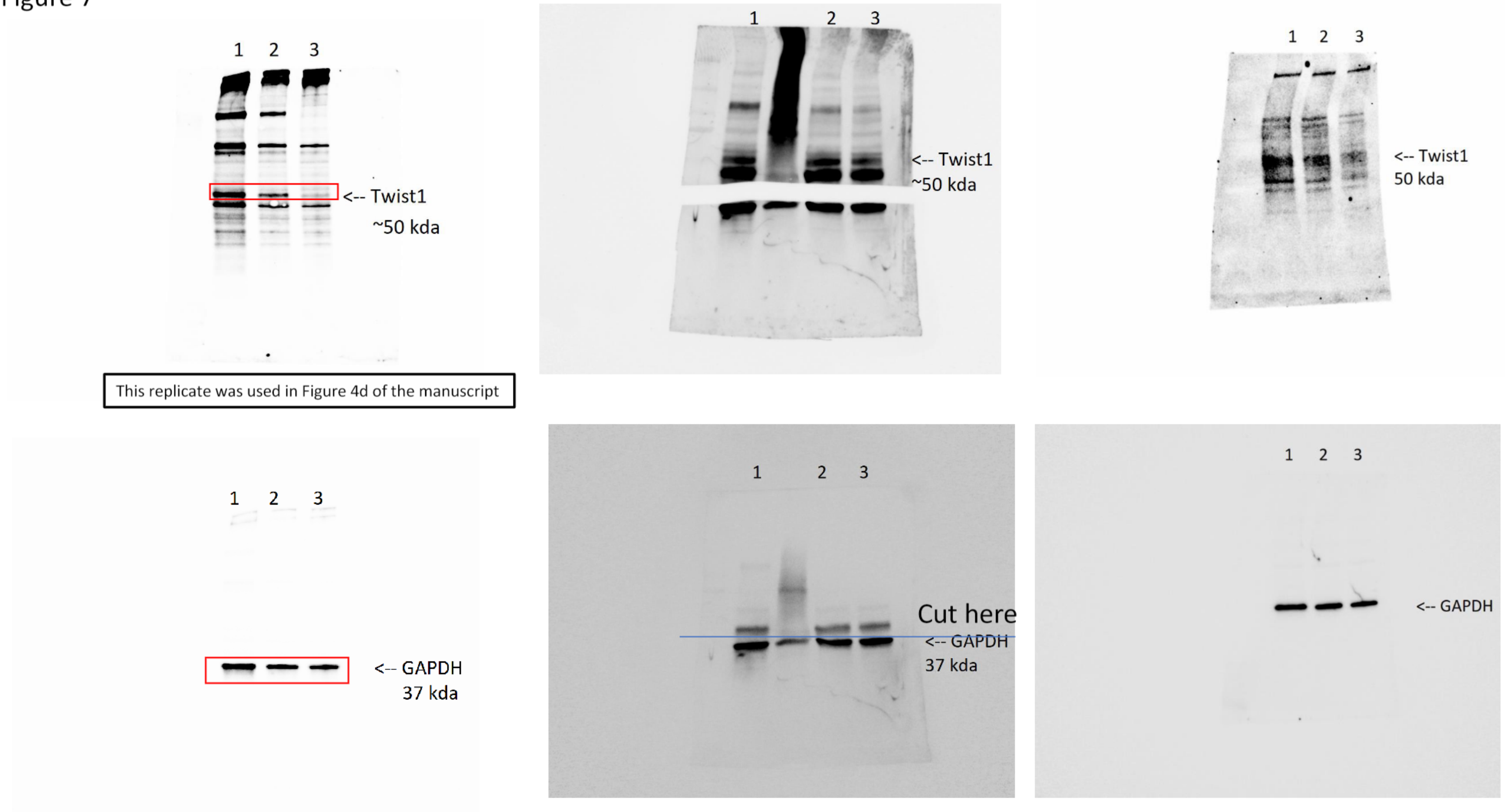

1 2 3
<-- Twist1
~50 kda
This replicate was used in Figure 4d of the manuscript
1 2 3
<-- Twist1
~50 kda
1 2 3
<-- Twist1
50 kda
1 2 3
<-- GAPDH
37 kda
1 2 3
Cut here
<-- GAPDH
37 kda
1 2 3
<-- GAPDH

Figure 8

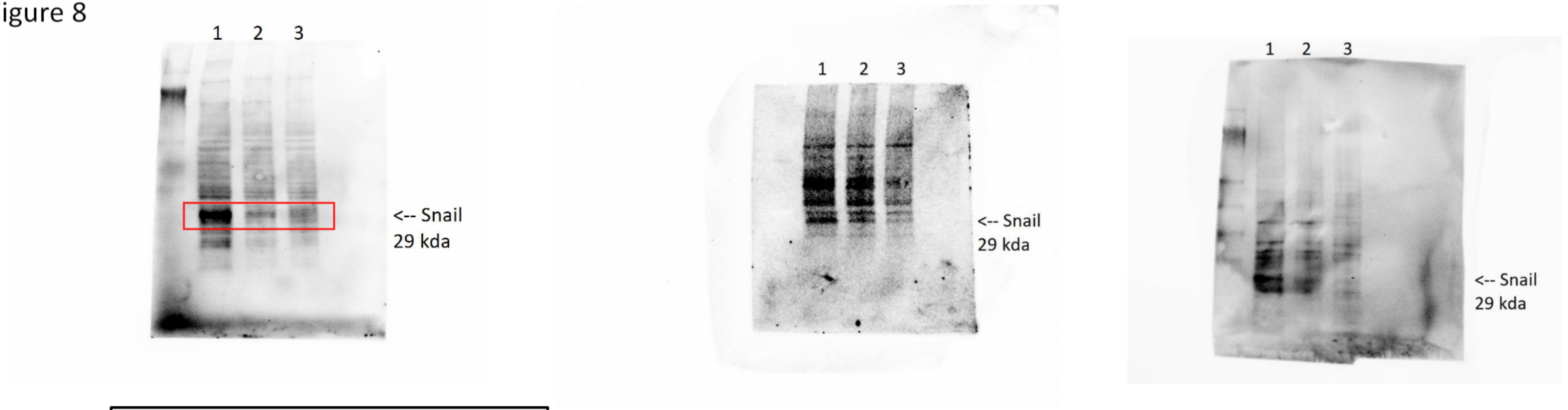

This replicate was used in Figure 4d of the manuscript

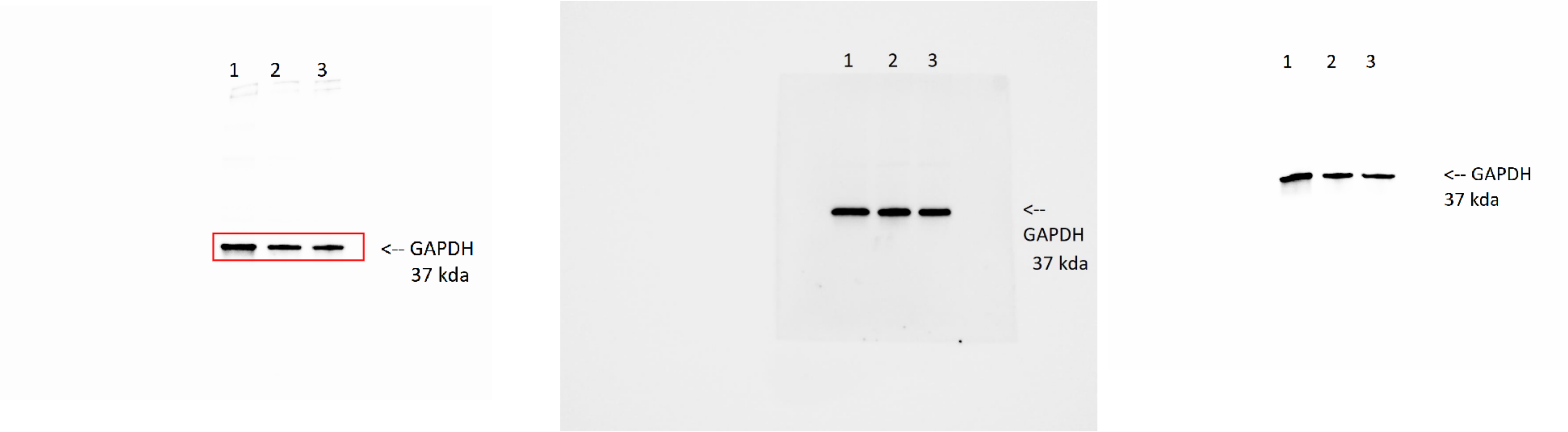

Raw western blots (Figures 9-13)

1. All lanes used in the manuscript and used for densitometry analysis (N=3) are labeled. Unlabeled lanes were not used for analysis.
2. All proteins used in the manuscript and used for analysis are appropriately labeled. Unlabeled proteins were either not used for analysis or were labeled in subsequent slides.
3. All protein lysate was obtained from DU145 cells.
4. All densitometry analysis was normalized to housekeeping protein (GAPDH).
5. Red boxes indicate how the blot was cropped for publication (approximately).
6. Labeling:
   - Lanes
     - 1: Control
     - 2: Strc 250 μM- 24h treatment
     - 3: Strc 250 μM- 48h treatment
   - Proteins
     - pGSK3b = phospho-GSK3β (ser9)
     - pAKT = phospho-AKT (ser473)

Figure 9

Membranes were cut approximately at 60-70 kda region before probing/imaging ROR1 and GAPDH.

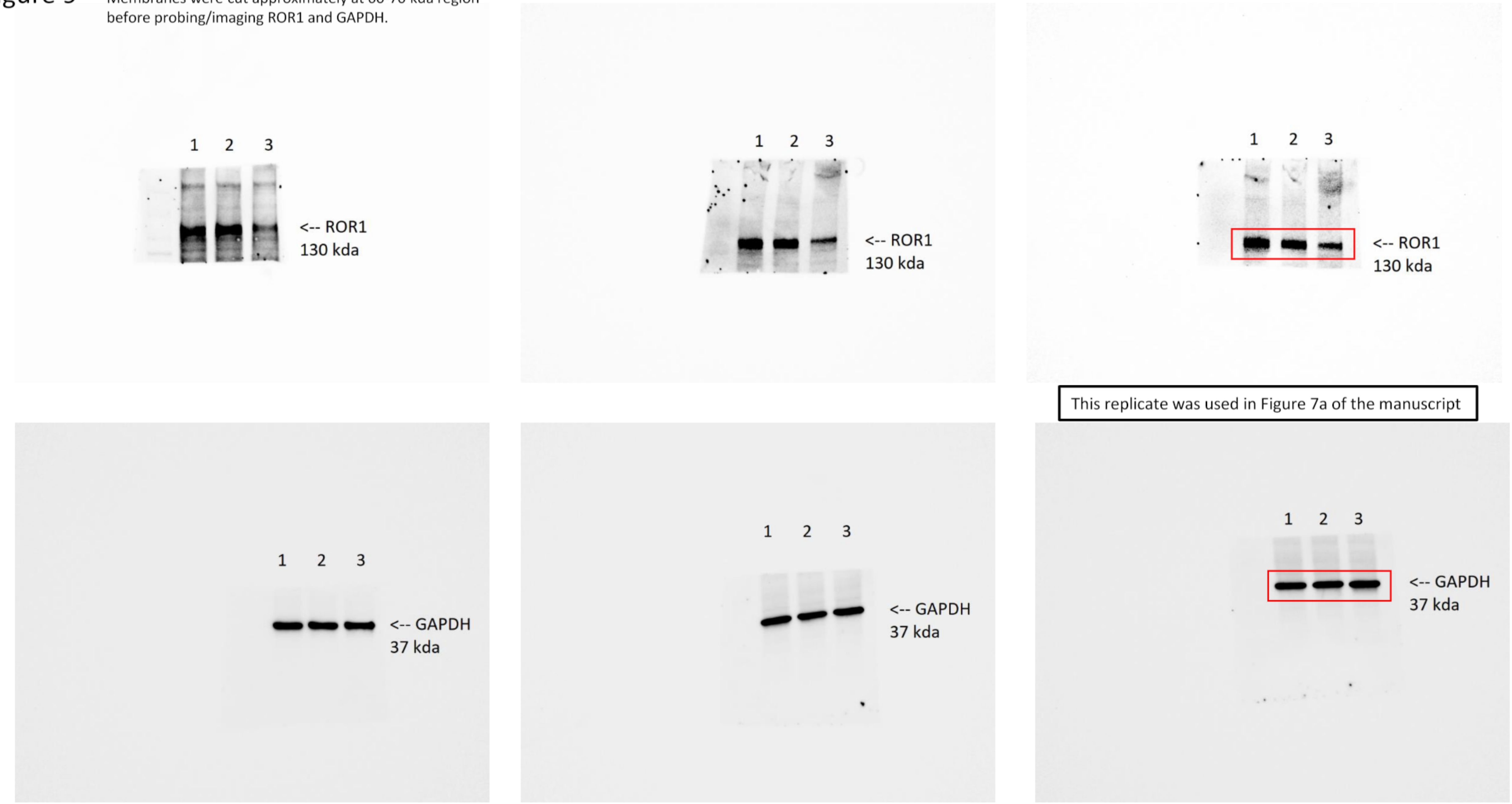

Figure 10

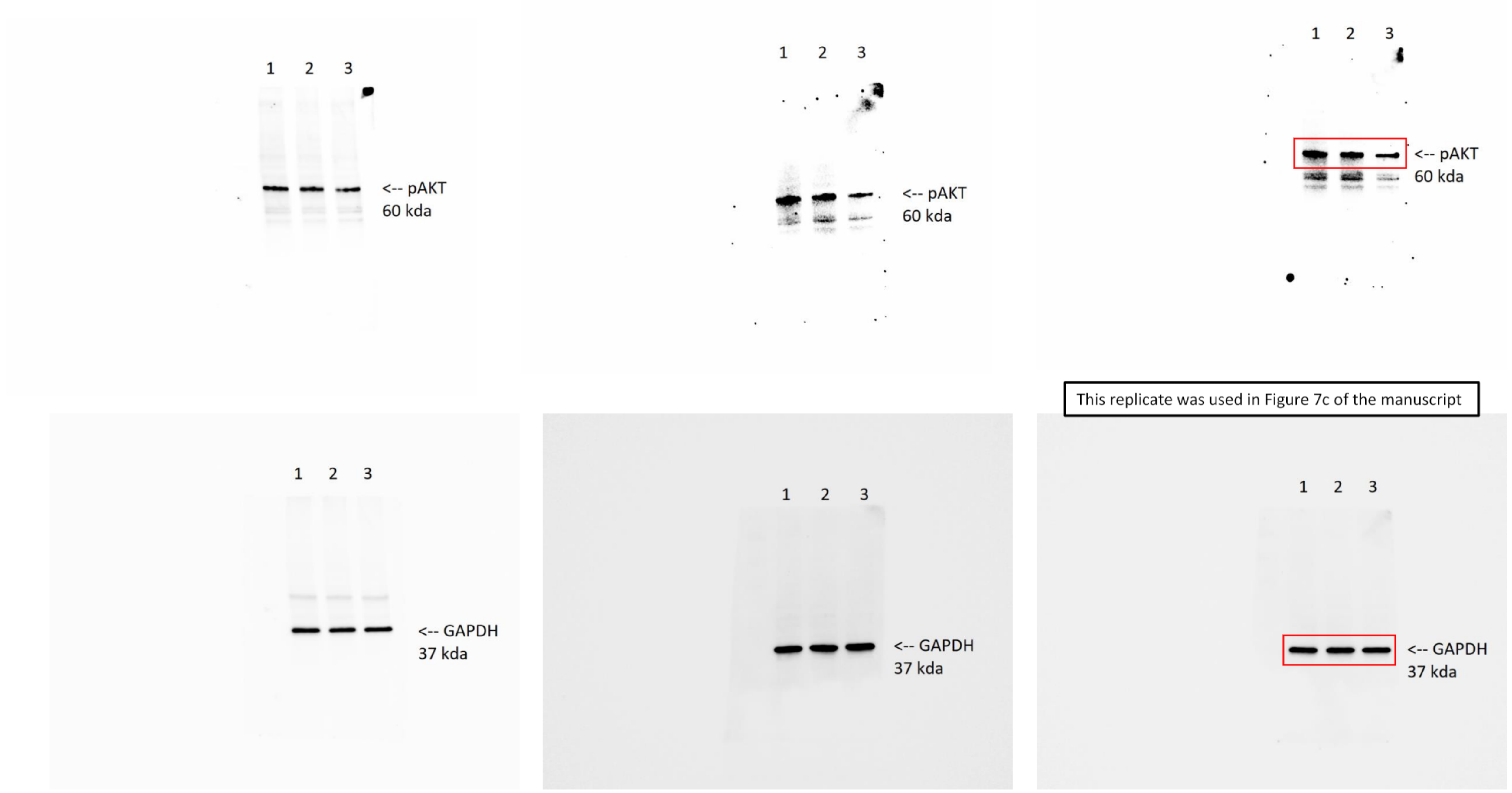
1 2 3
<-- pAKT
60 kda
1 2 3
<-- pAKT
60 kda
1 2 3
<-- pAKT
60 kda
This replicate was used in Figure 7c of the manuscript
1 2 3
<-- GAPDH
37 kda
1 2 3
<-- GAPDH
37 kda
1 2 3
<-- GAPDH
37 kda

Figure 11

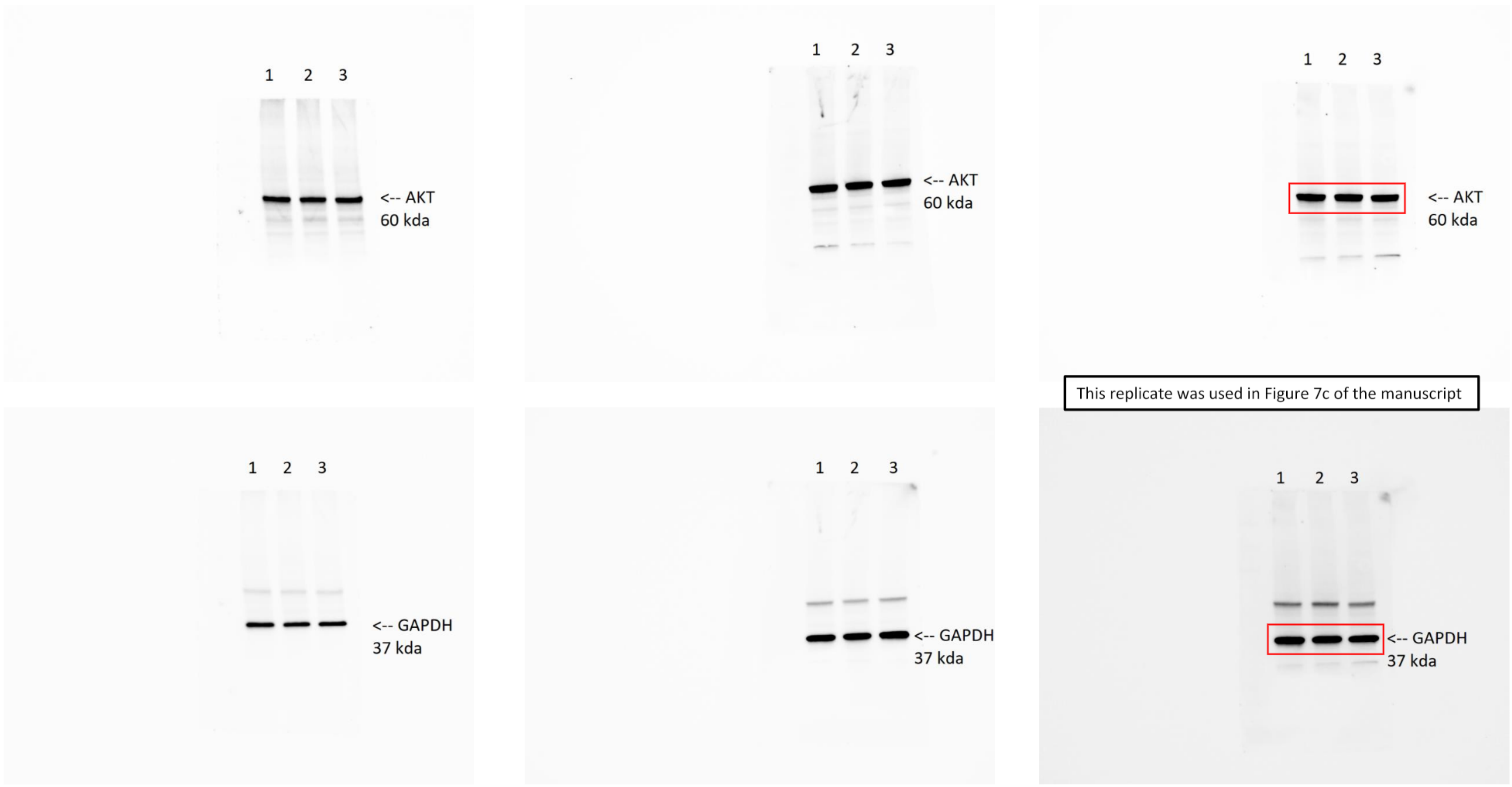
1 2 3
<-- AKT
60 kda
1 2 3
<-- AKT
60 kda
1 2 3
<-- AKT
60 kda
This replicate was used in Figure 7c of the manuscript
1 2 3
<-- GAPDH
37 kda
1 2 3
<-- GAPDH
37 kda
1 2 3
<-- GAPDH
37 kda

Figure 12

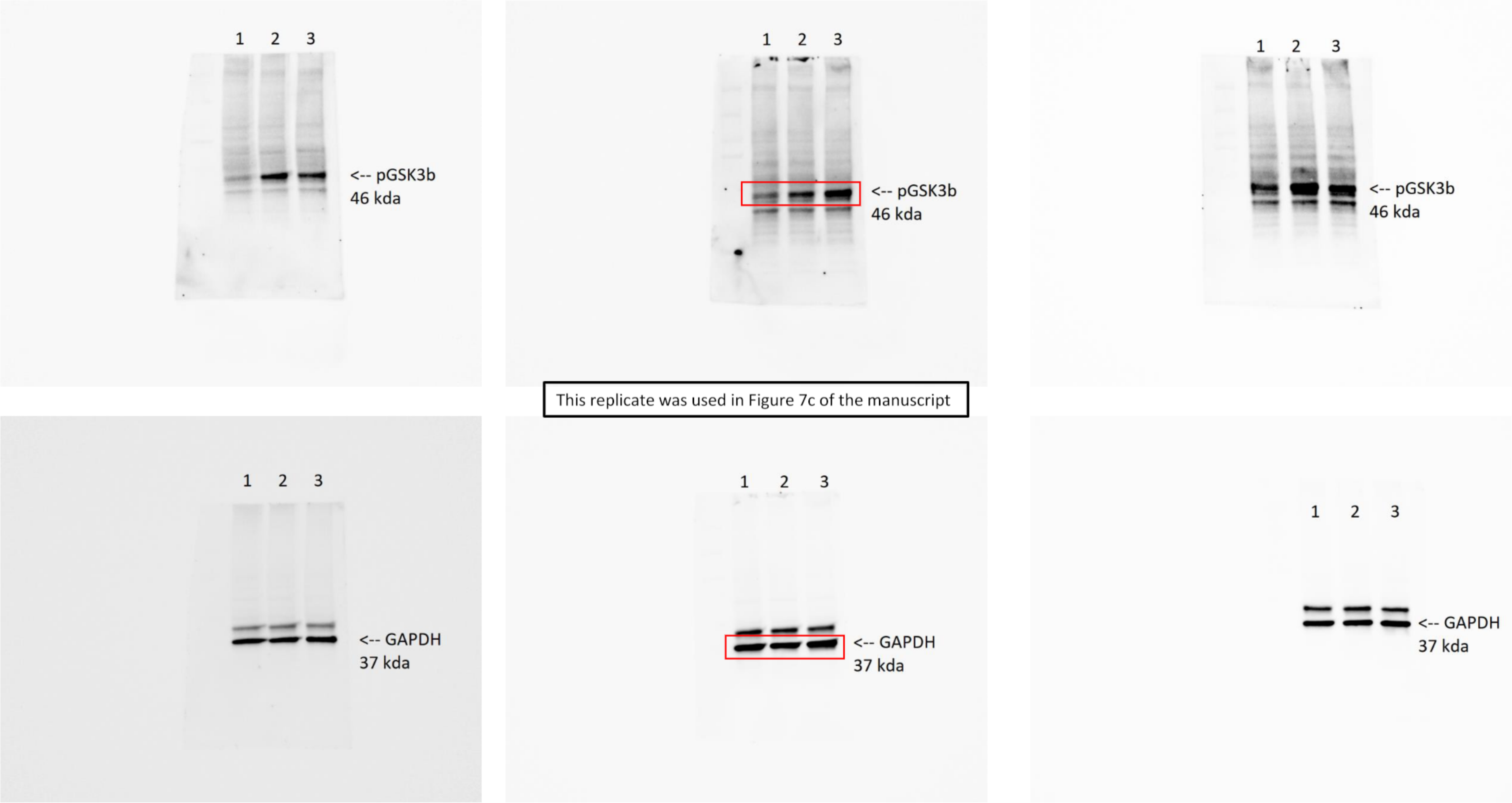

1
2
3
<-- pGSK3b
46 kda
1
2
3
<-- pGSK3b
46 kda
1
2
3
<-- pGSK3b
46 kda
This replicate was used in Figure 7c of the manuscript
1
2
3
<-- GAPDH
37 kda
1
2
3
<-- GAPDH
37 kda
1
2
3
<-- GAPDH
37 kda

Figure 13

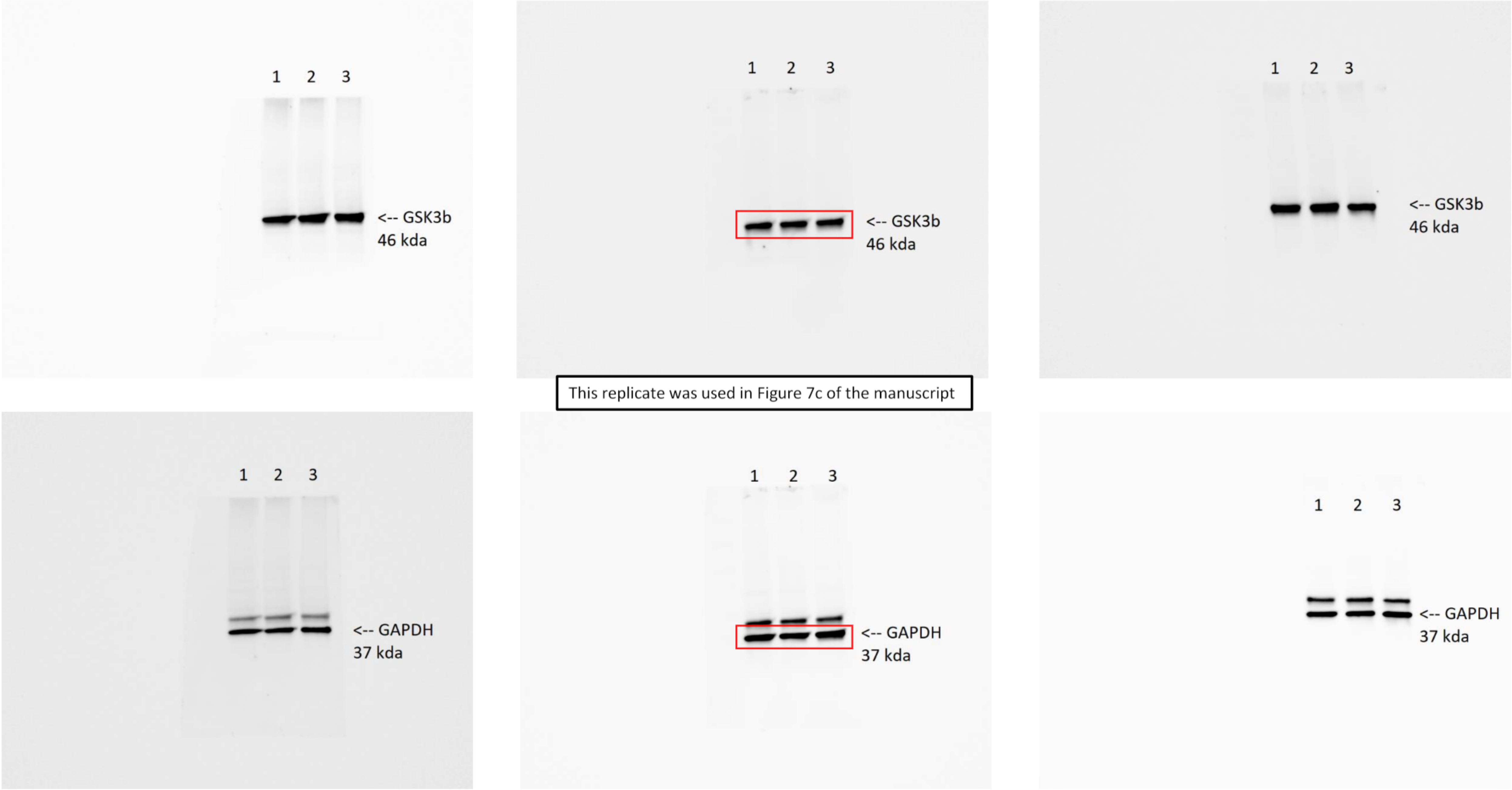
1 2 3
<-- GSK3b
46 kda
1 2 3
<-- GSK3b
46 kda
1 2 3
<-- GSK3b
46 kda
This replicate was used in Figure 7c of the manuscript
1 2 3
<-- GAPDH
37 kda
1 2 3
<-- GAPDH
37 kda
1 2 3
<-- GAPDH
37 kda
